# coTRaCTE predicts co-occurring transcription factors within cell-type specific enhancers

**DOI:** 10.1371/journal.pcbi.1006372

**Published:** 2018-08-24

**Authors:** Alena van Bömmel, Michael I. Love, Ho-Ryun Chung, Martin Vingron

**Affiliations:** 1 Department of Computational Molecular Biology, Max Planck Institute for Molecular Genetics, Berlin, Germany; 2 Department of Biostatistics, Department of Genetics, University of North Carolina at Chapel Hill, Chapel Hill, North Carolina, United States of America; 3 Otto Warburg Laboratory, Max Planck Institute for Molecular Genetics, Berlin, Germany; 4 Philipps-Universität Marburg, Fachbereich Medizin, Institut für Medizinische Bioinformatik und Biostatistik, Marburg, Germany; Johns Hopkins University, UNITED STATES

## Abstract

Cell-type specific gene expression is regulated by the combinatorial action of transcription factors (TFs). In this study, we predict transcription factor (TF) combinations that cooperatively bind in a cell-type specific manner. We first divide DNase hypersensitive sites into cell-type specifically open vs. ubiquitously open sites in 64 cell types to describe possible cell-type specific enhancers. Based on the pattern contrast between these two groups of sequences we develop “co-occurring TF predictor on Cell-Type specific Enhancers” (coTRaCTE) - a novel statistical method to determine regulatory TF co-occurrences. Contrasting the co-binding of TF pairs between cell-type specific and ubiquitously open chromatin guarantees the high cell-type specificity of the predictions. coTRaCTE predicts more than 2000 co-occurring TF pairs in 64 cell types. The large majority (70%) of these TF pairs is highly cell-type specific and overlaps in TF pair co-occurrence are highly consistent among related cell types. Furthermore, independently validated co-occurring and directly interacting TFs are significantly enriched in our predictions. Focusing on the regulatory network derived from the predicted co-occurring TF pairs in embryonic stem cells (ESCs) we find that it consists of three subnetworks with distinct functions: maintenance of pluripotency governed by OCT4, SOX2 and NANOG, regulation of early development governed by KLF4, STAT3, ZIC3 and ZNF148 and general functions governed by MYC, TCF3 and YY1. In summary, coTRaCTE predicts highly cell-type specific co-occurring TFs which reveal new insights into transcriptional regulatory mechanisms.

## Author summary

Differentiation of multicellular organisms into a variety of cell types with different morphology and function is the result of cell-type specific gene expression. The most important regulators of gene expression are transcription factors (TFs) binding to *cis*-regulatory sequences, such as enhancers or promoters. In particular, the combinatorial cooperativity of TFs is essential for defining the cell-type specificity. However, the experimental detection of cooperative TFs on a large scale is very difficult. Here, we develop a new strategy for predicting co-occurring TFs in a cell-type specific manner. We use a simple statistical test of TF co-binding between cell-type specific enhancers and globally active regulatory regions which assures the specificity and significance of predicted TF co-occurrences. Using chromatin accessibility data in 90 cell lines enables us to predict more than 2000 co-occurring TF pairs in 64 cell types. We confirm our predictions by multiple means, including comparison with large-scale experimental data. Based on our method, we obtain new insights into the cell-type specific TF cooperativity, and the complexity of transcriptional regulatory mechanisms.

## Introduction

In multicellular organisms, all cells carry the same genetic information, yet they differentiate during development into a variety of cell types with different morphology and function. This cell type differentiation is brought about by the execution of distinct gene expression programs. These programs, in turn, depend on regulatory arrangements accomplished by specific transcription factors (TFs), which bind to *cis*-regulatory sequences, such as enhancers or promoters [1]. *Cis*-regulatory elements are embedded in chromatin, whose basic repeating unit is the nucleosome. The presence or absence of these nucleosomes determines whether or not *cis*-regulatory elements are accessible for TF. Thus, accessibility of chromatin is a prerequisite for *cis*-regulatory elements to exert their regulatory effects. In eukaryotes, regulatory decisions are usually directed by a specific combination of TFs that act cooperatively rather than individually [2]. Therefore, the identification of cell-type specific cooperativity among TFs is a crucial step in understanding cell differentiation.

Cell-type specific co-operative binding of TFs has so far been primarily studied using groups of promoter sequences active in the cell type of interest [3–7]. In principle, the significance of such interactions can be tested by comparing the expected and observed number of co-occurrences of two motifs in selected promoters. Usually, the promoters considered are selected from among differentially expressed genes identified using gene expression data. In recent years, three approaches have been developed to investigate cell-type specific cooperativity between TFs in accessible chromatin regions. The first approach uses experimental data on TF binding determined by ChIP-seq or ChIP-chip for several TFs to detect significant co-occurrence among them. Typically the number of ChIP-seq peaks for two TFs co-occurring at a specific location is compared to the number of peaks for each individual TF [8, 9]. An alternative strategy integrates overrepresentation analysis of secondary motifs in peak regions bound by the primary TF [6, 8, 10–12]. Both strategies yield highly precise predictions but are restricted to TFs and cell types for which experimental data is available. The largest available human dataset is provided by the ENCODE project [13] and comprises several thousand ChIP-seq experiments. However, ChIP-seq experiments are available for only 87 distinct TFs in the five most studied cell lines [8]. The number of experiments in other cell lines is much smaller, with only a few distinct TFs represented.

The second approach to predict TF cooperativity in a cell-type specific manner combines gene expression measurements with the investigation of the regulatory regions of co-expressed genes for overrepresentation of TF motifs [5, 14]. For example, one previous study used a combination of DNA accessibility and gene expression data to build regulatory maps of Drosophila embryonic development [15]. The advantage of this approach is that gene expression data provides evidence of the functional effect of a specific combination of TFs. The disadvantage of this approach is that it can only be applied to the analysis of promoter sequences or to the small number of known enhancer-target gene pairs, because the target genes of distant regulatory regions are difficult to identify. Therefore this approach is also limited by the availability of experimental data.

The third approach to predict TF co-occurrence uses the experimental evidence of open chromatin derived from DNase-seq experiments to find significantly co-occurring pairs of TFs. Previous studies have focused on predicting direct TF-TF dimerization using fixed spacing and orientation of TF motifs [16–18]. Alternatively, the occupancy of binding motifs in DNaseI footprints can be investigated as this provides precise information on DNA-protein binding due to nonuniform DNaseI cleavage [19]. However, these methods require several thresholds to be specified in advance for the identification of the accessible chromatin regions or for the definition of binding motif hits and their spacing. Crucially, these methods do not focus on the cell-type specificity of the predicted TF cooperativity.

To address these limitations, here we propose a new method, coTRaCTE, for detecting pairs of TFs which preferentially co-occur in a cell-type specific manner. Our method incorporates two novel refinements which overcome the limitations of previous approaches. First, we consider accessible chromatin regions as determined by DNase-seq and divide the DNaseI-hypersensitive regions (DHSs) into those that are open in many cell-types (ubiquitously open DHSs, hereafter “ubiq-DHS”) and those that are accessible in a limited number of cell types only (cell-type specific DHSs, hereafter “CTS-DHS”). It is common practice to use such DHSs as a proxy for enhancer elements [20–22], in particular since promoters tend to be ubiquitously DNase-accessible [23]. The statistical advantage of contrasting the cell-type specific TF co-occurrences to the ubiquitous ones is in the usage of the ubiquitous sites as background sequences from which we can discriminate the TF co-occurring signal and from which we can assess the significance.

Using a large scale DNase-seq data set from the ENCODE project, we identify thousands of CTS-DHSs in 64 distinct cell types, which are likely to represent cell-type specific enhancers. The ubiq-DHSs represent chromatin that is constitutively open in all studied cell types. One advantage of coTRaCTE is that it does not require any thresholds to be defined for the identification of cell-type specific enhancers. The only user-defined parameter is the number of CTS-DHSs that should be taken into account for further analysis. The second advantage of coTRaCTE is that putatively cooperative TF pairs are assigned a statistical significance score based on an appropriate genomic background of open chromatin.

We apply coTRaCTE to all possible TF pairs represented among 554 TRANSFAC motifs identified in 64 cell types to produce an atlas of predicted co-occurring TF pairs within cell-type specific enhancers. Besides testing our method globally on 64 cell types, we present a more detailed local analysis of co-occurring TF pairs using embryonic stem cells (ESCs) as a study system. We decided to use ESCs as a proof of principle to assess our TF network predictions due to the availability of extensive experimental data for these cells. Over the past decade, the transcriptional regulatory network of embryonic stem cells (ESCs) has been intensively studied using various experimental techniques such as mass spectrometry [24], ChIP-chip and ChIP-seq data with microarray expression data [25] as well as bioinformatic techniques ([26, 27] for review). Although ESCs have been extensively investigated most studies have focussed on core pluripotent regulators such as OCT4, NANOG and SOX2 in addition to other known pluripotent regulators such as KLF4, DAX1, ESRRB, REX1 and c-MYC. Therefore other TFs that potentially cooperate with these core regulators remain to be investigated in detail. Our method allows us to investigate all TF pairs represented in the motif collection that are putatively cooperative in ESCs. Moreover, we reveal several striking differences in the predicted TF networks found in undifferentiated and differentiated ESCs.

## Materials and methods

### Cell type-specific chromatin accessibility

Currently, the standard approach to measure chromatin accessibility genome-wide is to digest chromatin with the endonuclease DNaseI followed by sequencing (DNase-seq). DNaseI is used to preferentially cleave accessible chromatin regions, which are therefore referred to as DNase hypesensitive sites (DHS). The DNase-seq experiment generates a genome-wide map of the accessible chromatin regions [28]; i.e. the greater the number of sequenced reads mapping to a certain region, the greater the sensitivity of that region to DNaseI digestion and therefore the greater the accessibility of its chromatin.

To determine hypersensitive regions which are most specific for individual cell types, we used data from 164 ENCODE experiments [13] across 88 healthy and 2 cancer cell lines. Biologically similar cell lines were grouped into one cell type, resulting in a total number of 64 cell types in our study. See S1 File for the exact grouping of all cell lines into cell types. Only healthy cell lines were analysed since we are interested in variations in chromatin accessibility determined by cell type identity rather than those determined by disease state or cell immortality. However, we did include two cancer cell lines (K562 and HeLa-S3) to facilitate comparison of our results with the large number of experimental studies analyzing these two cell lines. To account for the high technical variability among DNase-seq experiments from different research centers only experiments conducted in a single center (i.e. University of Washington) were considered for the analysis.

### Predicting cell-type specific enhancers

**Fig 1:**
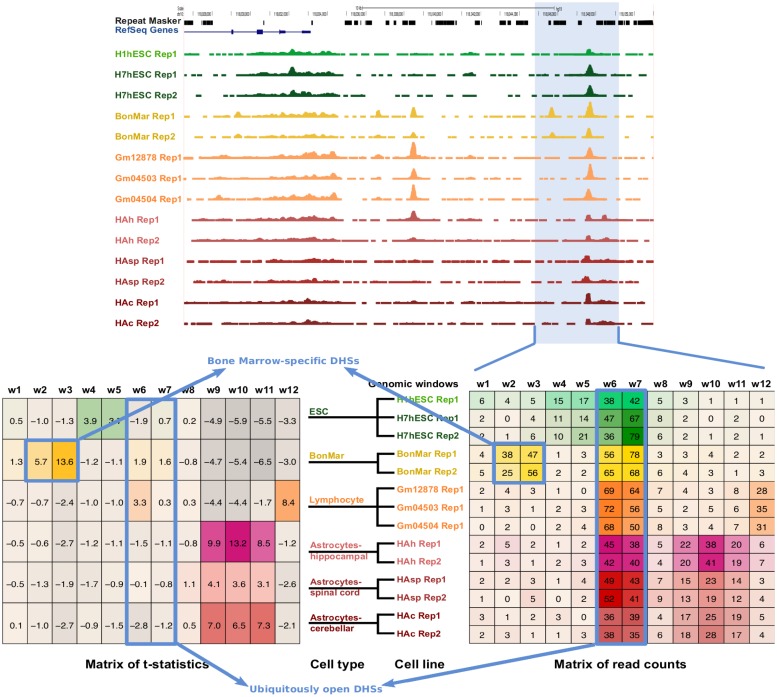
Overview of the method for the determining of cell-type specific DNase hypersensitive sites (CTS-DHSs). The top panel shows the raw DNase-seq data for 14 samples of 7 different cell lines (highlighted with different colors). Then, for each genomic window *w*1, …, *w*12, the log normalized read counts for each sample are calculated (matrix of read counts in the bottom right panel). The *t*-statistics are then calculated for each genomic window and each cell type over all corresponding cell lines (matrix of *t*-statistics in the bottom left panel). Windows *w*6 and *w*7 with large read counts in all cell types have small *t*-score over all cell types and are referred to as ubiquitously open DHSs. Windows *w*2 and *w*3 with large read counts in bone marrow have a large *t*-score in bone marrow only and are referred to as bone marrow-specific DHSs (e.g. CTS-DHSs).

To quantify the cell-type specificity of the DHSs, we calculated the *t*-statistic-based measure as described in [29]. First, the DNase-seq reads from all experiments were counted, and log counts plus one pseudocount were normalized for sequencing depth by multiplying the read counts for each sample by the mean read count over all samples divided by the sample’s mean read count. Here, the available replicates were treated as separate samples. Next, we created a large matrix of log normalized read counts over all 164 samples in 200bp non-overlapping windows (Fig 1, bottom right panel) along the human genome (hg19 Ensembl assembly from genome.ucsc.edu) excluding strong repeat sequences. For each window *w*, $X_{i}^{w}$ denotes the log read count for sample *i* ∈ {1, …, *n*}. The set of all samples belonging to a given cell type *ct* ∈ {1, …, *m*} is denoted as *C*_*ct*_, i.e. *C*_*ct*_ ⊆ {1, …, *n*}. We assume that each sample *i* belongs to exactly one cell type, so that *C*_*ct*_ are pairwise disjunct. Denoting the cardinality of *C*_*ct*_ as *n*_*ct*_, the average DNase-seq profile for cell type *ct* is defined as ${\bar{X}}_{ct}=1/n_{ct}\sum_{i\in C_{ct}}X_{i}$. Thus, the global DNase-seq profile of all cell types is then: $\bar{X}=1/m\sum_{ct=1}^{m}{\bar{X}}_{ct}$. The unbiased cell-type variance is then given by: $s_{ct}^{2}=\frac{1}{n_{ct}-1}\sum_{i\in C_{cj}}{\left(X_{i}-{\bar{X}}_{ct}\right)}^{2}$. Assuming equal variance among all cell types, pooled within-class standard deviation is defined by:

$$\begin{array}{c} s=\sqrt{\frac{\sum_{j=1}^{m}\left(n_{j}-1\right)s_{j}^{2}}{\sum_{j=1}^{m}\left(n_{j}-1\right)}}=\sqrt{\frac{\sum_{j=1}^{m}\sum_{i\in C_{j}}{\left(X_{i}-{\bar{X}}_{j}\right)}^{2}}{\sum_{j=1}^{m}\left(n_{j}-1\right)}}\;. \end{array}$$

Then, we weight the differences from the global profile to the cell-type profile by the pooled standard deviation. This provides a *t*-statistic for cell type *ct* defined as:

$$\begin{array}{c} t_{ct}=\frac{{\bar{X}}_{ct}-\bar{X}}{\sqrt{1/m+1/n_{ct}}\cdot \left(s+s_{0}\right)}\;, \end{array}$$

with *s*_0_ denoting the mean of *s* over all windows to prevent division by small within-cell-type variance estimates. This calculation is then repeated for each genomic window *w* (see Fig 1), so that the *t*-statistic measures the corresponding cell-type specificity of each DHS.

An illustrative example for 14 samples from 7 different cell lines corresponding to 6 different cell types is shown in Fig 1. The top panel shows the raw DNase-seq tracks of 14 samples. The bottom right panel zooms into a small genomic region with 12 windows showing the matrix of the log-normalised read counts for all 14 samples. Then, all 14 samples originating from 9 cell lines are grouped into 6 cell types and the *t*-statistic is calculated for each window in each cell type (matrix shown in bottom left panel). Windows 6 and 7 have large read counts in all cell types thus their *t*-score is small in all cell types and they are referred to as ubiquitous DHSs. Windows 2 and 3 have large read counts in bone marrow thus their *t*-score is large in bone marrow only and they are referred to as bone marrow-specific DHSs.

Genomic regions with the largest positive *t*-score in each cell type are hereafter referred to as “cell-type specific DNase hypersensitive sites” (or CTS-DHSs). In contrast, ubiquitously open regions with global *t*-score close to zero are hereafter referred to as “ubiquitous DHSs” (or ubiq-DHSs). In constrast to other methods which identify DHSs that are open specifically in given cell type, the *t*-based measure accounts for within-cell-type variability of the DNase-I sequencing counts. This is a crucial feature of our method since it produces a ranking of genomic sites that are consistently hypersensitive in a given cell type, relative to an average profile of all studied cell types.

Read counting and genomic range manipulation were performed using BEDTools [30]. The *t*-statistic was calculated within the R statistics environment, using the sparse matrix package Matrix.

### Annotating DHSs with transcription factor binding affinity

**Fig 2:**
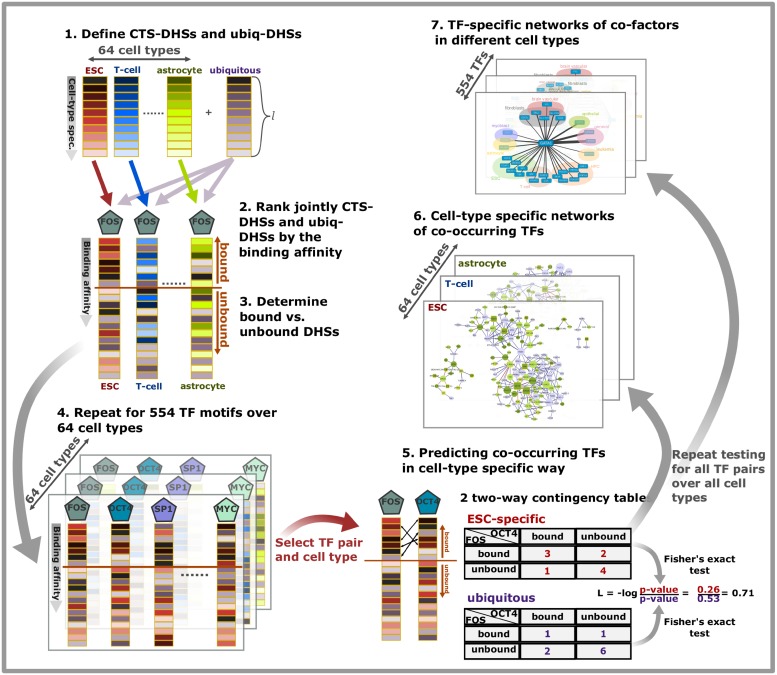
Schematic of the method for the detection of TF overrepresentation and co-occurrence in the cell-type specific DNase hypersensitive sites (CTS-DHSs). First, the *l*-most cell-type specific DHSs and the *l*-most ubiquitous DHSs are determined (1). Then, for each cell type separately, and for each TF of interest, CTS-DHSs and ubiquitous DHSs are jointly ranked by the binding affinity (2). The preset cutoff determines bound and unbound DHSs (3). This approach is repeated for all TF motifs and all investigated cell types (4). Co-occurring TF pairs on CTS-DHSs are predicted from the log score of *p*-values derived from two contingency tables with Fisher’s exact test (5) and summarized in cell-type specific TF networks (6) or in TF-specific networks with co-occurring partners in different cell types (7).

To predict bound and unbound sites for each particular TF in our study, we use the TRanscription factor Affinity Prediction method TRAP [31]. We first select the top-*l* cell-type specific CTS-DHSs of each cell type and the top *l* ubiq-DHSs. Then, for each TF motif of interest, its binding affinity to these sites is estimated using TRAP. TRAP quantifies TF binding using a biophysical model that produces binding affinity values for each TF motif to the particular DHS. This approach is superior to hit-based motif screening algorithms which use a threshold to distinguish between binding sites and non-binding sites. Notably, hit-based methods may fail to consider low-affinity binding sites, which might be essential for cell-type specific gene regulation [32, 33]. For each individual TF and each cell type, we use the binding affinity prediction for each DHS to rank the selected CTS-DHSs and ubiq-DHSs jointly by their predicted binding affinity for the given TF. DHSs ranked among the top-*k* are considered to be “bound” and all other DHSs are considered to be “unbound”. Considering the example in Fig 2(steps 1 and 2), we first take the FOS motif and select the ESC cell type (highlighted in red). Then, the *l* = 10 most *ESC*-specific DHSs (in red) and *l* = 10 most *ubiquitous* DHSs (in grey) are taken and jointly ordered by FOS predicted binding affinity. The same procedure is then repeated for *T-cell* (in blue), *astrocytes* (in green) and all other cell types. Then, the top *k* = 7 sites in each list are considered as bound by FOS, the remaining 13 sites are considered as unbound. The same scheme is then repeated for all other TFs (OCT4, SP1, MYC, etc).

For our analysis a list of 554 known TF motifs obtained from TRANSFAC 2012 database from BIOBASE Corporation ([34], www.biobase-international.com) was used. However, the TRANSFAC database contains redundant entries, since transcription factors are known to recognize more than one consensus sequence [35]. On the other hand, similar DNA sequences can be recognized by different TFs [36], thus different TFs might have the same motifs in the database. Therefore, we assigned the set of 554 TF motifs (hereafter “TF motifs”) to 306 individual TFs or TF groups/families (hereafter “TFs”), using the information provided by the TRANSFAC database and by [11], see S2 File. The calculation of TRAP affinities was done using the TRAP command line tool, the sorting and data manipulation was conducted within the R statistics environment.

### Co-occurrence of TFs within cell-type specific enhancers

To predict pairs of co-occuring TFs in a cell-type specific manner, we quantify (i) the degree of overlap between cell-type specific DHSs (corresponding to enhancers) bound by both TFs and (ii) the degree of overlap between ubiquitous DHSs bound by both TFs. To this end, first, we build two two-way contingency tables: one table for the co-binding of the TF pair in the cell-type under study, and the other table for the co-binding of the TF pair in ubiquitous DHSs. Then, the log ratio of *p*-values from the two contingency tables is calculated to estimate the likelihood that a TF-pair co-occurrence is cell-type specific rather than ubiquitous.

Technically, we define two binary variables *X* and *Y* identifying the existence of a binding motif in a particular DHSs for the first TF and for the second TF in a pair, respectively. For the top *k* DHSs having the highest predicted affinity for the first TF (“TF_1_”), the binary variable *X* equals one and we defined these DHSs as bound by the first TF. Correspondingly, for the top *k* DHSs having the highest predicted affinity for the second TF (“TF_2_”), the binary variable *Y* equals one, thus these DHSs are bound by the second TF. Formally for each DHS_*i*_, where *i* = 1, …, 2*l*:

$$\begin{array}{ccc} X(i) & = & \left\{ \begin{array}{cc} 1 & {\text{DHS}}_{i}\;\text{bound}\;\text{by}\;{\text{TF}}_{1}\\ 0 & \text{otherwise} \end{array}\right.\\ Y(i) & = & \left\{ \begin{array}{cc} 1 & {\text{DHS}}_{i}\;\text{bound}\;\text{by}\;{\text{TF}}_{2}\\ 0 & \text{otherwise.} \end{array}\right. \end{array}$$ (1)

The third binary variable *Z*_*ct*_ indicates cell-type specific DHSs for a particular cell type *ct* and is defined as follows:

$$\begin{array}{c} Z_{ct}\left(i\right)=\{\begin{array}{cc} 1 & {\text{DHS}}_{i}\;\text{is}\;\text{cell-type}\;\text{specific}\;\text{for}\;\text{cell}\;\text{type}\;ct\\ 0 & {\text{DHS}}_{i}\;\text{is}\;\text{ubiquitous}\;. \end{array} \end{array}$$ (2)

Then two individual *X*, *Y*-tables stratified by *Z*_*ct*_ according to cell type can be constructed, as shown in Table 1. Due to the selection of the top *l* cell-type specific DHSs and the top *l* ubiquitous DHSs, both of the tables have the same size *l* and are therefore simply comparable.

**Table 1:** Two contingency tables for co-occurrence of two TFs on CTS-DHSs (top) and on ubiq-DHSs (bottom).

cell-type specific: *Z*_*ct*_ = 1			
	DHSs bound by *TF*_2_	DHSs unbound by *TF*_2_	∑
DHSs bound by *TF*_1_	*n*_11*ct*_	*n*_10*ct*_	*n*_1+*ct*_
DHSs unbound by *TF*_1_	*n*_01*ct*_	*n*_00*ct*_	*n*_0+*ct*_
∑	*n*_+1*ct*_	*n*_+0*ct*_	*n*_++*ct*_ = *l*
ubiquitous: *Z*_*ct*_ = 0			
	DHSs bound by *TF*_2_	DHSs unbound by *TF*_2_	∑
DHSs bound by *TF*_1_	*n*_11*u*_	*n*_10*u*_	*n*_1+*u*_
DHSs unbound by *TF*_1_	*n*_01*u*_	*n*_00*u*_	*n*_0+*u*_
∑	*n*_+1*u*_	*n*_+0*u*_	*n*_++*u*_ = *l*

The independence of both variables *X* and *Y* can be assessed using Fisher’s exact test (FT) and then compared with respect to variable *Z*, i.e. in the cell-type specific case and in the ubiquitous case. To quantify the difference between the two tables, we define a score *L* as the log ratio of the *p*-value obtained from FT in the cell-type specific table and of the *p*-value obtained from FT in the ubiquitous table. Formally, the *L*_*ct*_ score for cell type *ct* is defined as a log ratio of the probability that the expected counts *m*_*ct*_ of CTS-DHSs bound by both TFs are larger than the observed value *n*_11*ct*_ and of the probability that the expected counts *m*_*u*_ of ubiquitous DHSs bound by both TFs are larger than the observed value *n*_11*u*_:

$$\begin{array}{c} L_{ct}=-\log[\frac{P(m_{ct}\geq n_{11ct})}{P(m_{u}\geq n_{11u})}]\;. \end{array}$$ (3)

With this definition, the *L*_*ct*_ score contrasts the likelihood of co-occurrence of both TFs on the cell-type specific sites with the likelihood of their co-occurrence on ubiquitous sites. The larger the *L*_*ct*_ score, the greater the association between the two TFs on the CTS-DHSs relative to the ubiquitous DHSs. Thus, the ubiquitously open chromatin regions serve as background model to assess the significance of the cell-type specific co-occurrence. The *L*_*ct*_ score is computed for all possible TF pairs in each cell type of interest. Thus TF pairs with the largest positive *L*_*ct*_ score are more likely to co-occur in the particular cell type than in ubiquitously open chromatin and are predicted as co-occurring TFs in a cell-type specific manner. Moreover, TF pairs with the largest negative *L*_*ct*_ score are TF pairs which co-occur generally on the ubiquitous DHSs and not in a cell-type specific way. We refer to these as ubiquitously co-occurring TF pairs.

The method described above is summarized in Fig 2(Step 5) using the TF pair FOS and OCT4 for illustration. The binding affinity of these TFs is predicted for 10 ESC-specific and 10 ubiquitous DHSs. Selecting the top *k* = 7 DHSs as bound, the two contingency tables are derived and their significance is assessed with Fisher’s exact test. The log score ratio *L* compares the significance of the joint binding on CTS-DHSs by FOS and OCT4 to the significance of the joint binding on ubiquitous DHSs by this TF pair.

After evaluation of various combinations of the parameters *k* and *l* (see S1 Appendix and S2 Fig for more details), the following combination, which resulted in the most consistent results, was selected for the prediction of co-occurring motifs: *k* = 1000 (i.e. the top 1000 DHSs ordered by binding affinity are considered as “bound”) and *l* = 5000 (i.e. a total of 5000 CTS-DHSs and 5000 ubiq-DHSs are analyzed). With 5000 cell-type specific DHSs, the overlap of DHSs between different cell types is still very low and this is why we generally recommend this setting. The condition of low DHS overlap can also be verified on a new data set and the parameter changed accordingly. The number of 1000 “bound” DHSs has always worked well in our experience. The alternative of systematically testing this cut-off in search for the most statistically significant results seems both overly computationally demanding and the mere number of tests may make it hard to find statistically significant results.

Testing all possible TF motifs among different TF groups results in a total of 111241 TF pairs in each of 64 cell types, corresponding to total number of 14239 × 10^6^ tests. The obtained *p*-values from the Fisher’s exact test were corrected for multiple testing using the Benjamini-Hochberg method [37] by considering each cell type separately.

The complete contingency table analysis and statistical testing were realized within the R statistics environment using the log-linear models of MASS package [38]. Figures were created within R using packages ggplot2 [39] and circlize [40] and the networks were created using Cytoscape [41].

### TF motifs overrepresented in cell-type specific enhancers

Using our general study design allows us to investigate not only the co-binding TF pairs but the overrepresented TF motifs in the cell-type specific enhancers. To this end, we constructed a single two-way-contingency table for each TF and for each cell type. The row variable *X* distinguishes the bound DHSs from the unbound, whereas the column variable *Z* distinguishes the cell-type specific DHSs from the ubiquitous DHSs. The independence of variables *X* and *Z* can be assessed using Fisher’s exact test. TF motifs with the highest significance are considered as overrepresented TF in the particular cell type. The overrepresentation analysis was conducted within the R statistics environment.

### Validation of overrepresented TF in cell types

Gene and protein functions were determined using the Entrez Gene database [42, 43, www.ncbi.nlm.nih.gov/gene], UniProt Knowledgebase [44, www.uniprot.org] and the GeneMANIA tool [45, www.genemania.org/]. Expression analysis of TFs in various cell types was derived from Ensembl [46] and from GTEx [47].

## Results

### CTS-DHSs correspond mainly to enhancers whereas ubiq-DHSs do not

To investigate their genomic location, we selected the 5000 highest scoring cell-type specific sites (CTS-DHS) for each cell type and the 5000 highest scoring ubiquitous sites (ubiq-DHS) across all cell types according to a *t*-statistic based measure. We found that the large majority (88%) of the CTS-DHSs are located in intronic and intergenic regions whereas only 8% are situated in promoters (defined as the region starting 5000bp upstream of an annotated TSS) and < 4% overlap with annotated exons (hg19 Ensembl assembly, Release 75 from genome.ucsc.edu). The only exception is the primary T-cell for which 19% of CTS-DHSs were located in exons and 22% were located in promoters. In contrast, the genomic distribution of ubiq-DHSs differs markedly from that of the CTS-DHSs, with 43% of ubiq-DHSs overlapping promoter regions (see S3 Fig). Further, we investigated the GC content of the two types of DHSs. The mean GC content of the CTS-DHSs varies between 39% (cardiac atrial fibroblast and spinal cord astrocytes) and 63% (primary T-cell). However, the majority of CTS-DHSs has a mean GC content below 50%, whereas the mean GC content of the ubiq-DHSs lies much higher by 58% (see S9 Fig). Our findings suggest that the CTS-DHSs correspond mainly to cell-type specific enhancers, a conclusion supporting previous studies that analyzed a different data set [23, 29] or analyzed specific cell lines [20, 22, 48, 49].

### Individual TFs overrepresented within cell-type specific enhancers confirm previous findings

As a first test of the power of our approach, we considered to what extent our observations for individual TFs recapitulate previous findings. To this end, we investigated individual TFs that are overrepresented within the identified CTS-DHSs representing cell-type specific enhancers. We expected these individual TFs to include only a subset of the transcription factors important for cell-type specificity. Notably, overrepresented TF motifs within CTS-DHSs are motifs having the highest significance (i.e., smallest *p*-value) of the Fisher’s exact test in the particular cell type.

We identified a high confidence set of individual TFs consisting of the 50 most significant TFs binding accessible chromatin in each studied cell type. Within this set, we identified 23 TFs that were observed to be among the most significant TFs occurring in at least 30 out of 64 cell types. These 23 TFs are generally enriched within the CTS-DHSs compared to ubiq-DHSs, regardless of cell type. This observation confirms recently published findings of [50] describing several characteristics common to cell-type specific chromatin accessible regions. Most of the TFs enriched in CTS-DHSs are known regulators of many multiple genes and are primarily involved in general cellular functions such as (i): apoptosis, energy metabolism or cellular growth (HIF1, NRF1, SP1), (ii) cell cycle functions (E2F, MYC, MAX) or (iii) in general development of organs (CREB1, TFAP2A, TFAP2C, EGR family, KLF4, HIC1, TEAD2). The fact that these TFs are very important transcriptional regulators of general cellular functions is consistent with their enrichment in the majority of CTS-DHSs. Taken together, these findings suggest that individual TFs overrepresented within CTS-DHSs of multiple cell types perform general cellular functions.

#### Overrepresented TFs on cell-type specific enhancers have specific functions

Among individual TFs overrepresented in CTS-DHS we found sets of TFs which were specifically enriched in the CTS-DHSs of either one particular cell type or of functionally related cell types. Many of these specifically enriched TFs have known functions in the related cell type. For example, AP1, FOS, JUN, BACH proteins and ZEB1 are enriched in cell types of the immune system; TCF3, MYF, MYOD and MYOG in myoblasts; STAT4, STAT6, NFATC, IKZF2, SOX2 and SOX3 in blood vessel; TAL1, TCF3 and ZNF238 in dermal and pulmonary fibroblast; FOX factors in lung cell types and RUNX factors in astrocytes. For detailed information about the overrepresented factors in all cell types see S1 Appendix, S1 Table and S4 Fig. Taken together, these findings suggest that individual TFs overrepresented within CTS-DHSs of a single cell type (or a small number of related cell types) perform specific cellular functions.

### Predictions of co-occurring TF pairs generated from cell-type specific open chromatin regions are cell-type specific

Significantly co-occuring TF pairs were defined as pairs with the *L*_*ct*_ score larger than the 99.5%-quantile of the empirical distribution of all *L*_*ct*_ scores in the particular cell type (see Materials and methods). In this way, we predicted a total of 5 257 co-occurring pairs of TF-motifs within the identified CTS-DHS. These significant TF-motif pairs were then assigned to their corresponding pairs of TFs, resulting in a total of 2 359 significant TF pairs. To test whether the identified co-occurring TFs are cell-type specific, we investigated the overlap of the predicted sets of co-occurring TFs between all pairs of cell types.

**Fig 3:**
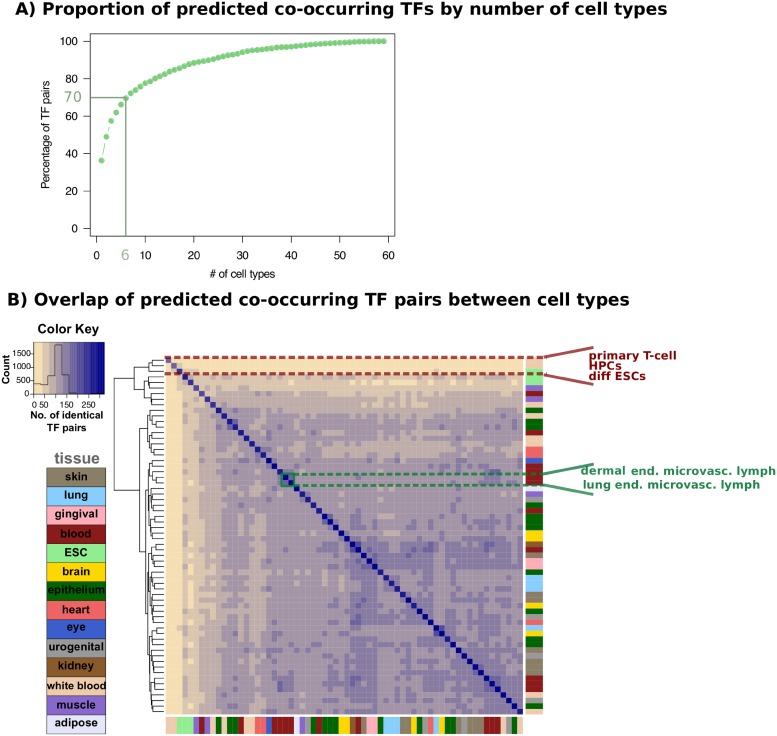
Cell-type specificity of predicted co-occurring TF pairs by CoTRaCTE. A) Cumulative plot of number of cell types in which TF pairs are predicted to co-occur. As highlighted with the green lines, 70% of all predicted co-occurring TF pairs are highly cell-type specific i.e. they are observed in only 6 cell types or fewer. B) Heatmap of overlapping predicted co-occurring TF pairs over 64 cell types. Each cell depicts the number of TF pairs shared between the corresponding pair of cell types. Primary T-cell, HPCs and ESCs (highlighted with red dashed lines) possess sets of co-occurring TF pairs which are distinct from those of other cell types. Functionally related cell types such as microvascular endothelial dermal lymph cells and microvascular endothelial lung lymph cells (highlighted with green dashed lines) share a large number of TF pairs.

We found that the majority of the predicted TF pairs shows a high degree of cell-type specificity: 1641 (70%) of the co-occurring TF pairs are found in 6 or fewer cell types, of which 856 (36%) are found in one cell type only (see Fig 3A) confirming the cell-type specificity of our predictions. As expected, highly related cell types originating from the same tissue showed partial overlaps between the sets of their predicted co-occurring TF pairs. For example, microvascular endothelial dermal lymph cells share 65% of their co-occurring TF pairs with microvascular endothelial lung lymph cells, which are closely related cells both morphologically and functionally. Interestingly, primary cell types such as primary T-cell, hematopoietic progenitor cells (HPCs) and embryonic stem cells (ESCs) possess very distinct sets of co-occurring TF pairs compared to all other differentiated cell types, see Fig 3B. For example, primary T-cells share a maximum of only 12% of their co-occurring TF pairs with HPCs and only 6% with other T-cells. Similarly, differentiated ESCs share a maximum of only 24% of their co-occurring TF pairs with undifferentiated ESCs and only 18% with other ES cell lines. These observations suggest that primary cell types and differentiated cell types differ substantially not only in their sets of co-occurring TF pairs but also in their CTS-DHSs.

On the other hand, we identified 158 co-occurring TF pairs common to at least 30 out of 64 cell types. These common TF pairs include mainly homeobox factors (ALX1, POU2F1, ONECUT, HNF1, homeodomain NKX factors) and members of the forkhead-box (FOX) family (see S5 Fig) which have general functions in cellular and organismal development and cell differentiation [42–44, 51, 52]. This finding confirms observations describing partial sequence similarity of cell-type specific open chromatic regions [50]. Our results suggest that the CTS-DHSs are enriched for pairs of homeobox and forkhead-box binding motifs and for a large number of highly cell-type specific TF pairs.

Further, we compared the co-occurring TF pairs with the individual TFs overrepresented on the CTS-DHSs described above. For all cell types, co-occurring TF pairs are not just combinations of the single TFs, moreover the co-occurring TF pairs include more cell-type specific TFs such as KLF4 and cMYC in ESCs or tumor-related genes STAT5 and TAL1 in leukemia. We conclude that the TF pairs predicted by CoTRaCTE contribute additional information on top of the single TF overrepresentation analysis.

### Predictions of co-occurring TF pairs generated from ubiquitously open chromatin regions are highly consistent across cell types

Selecting TF pairs with the smallest *L*_*ct*_ scores (smaller than the 0.5%-quantile) over all cell types identifies TF pairs that preferentially co-occur in ubiq-DHSs rather than in CTS-DHSs (see S1 Appendix and S6 Fig). The ubiquitously co-occurring TF pairs are derived for each cell type separately, resulting in 64 distinct sets of ubiquitous TF pairs. Notably, these sets of ubiquitous TF pairs are almost identical regardless of which cell type was employed to generate them (see S8 Fig). This finding supports our claim that cell-type specific TF cooperativity can be detected only when genomic regions of interest are contrasted with an appropriate genomic background having the same chromatin accessibility. Further, the ubiquitous TF pairs include several TFs, such as ATF, CREB, E2F1, NFY, NRF1, SP1, TP and STAT factors which have previously been described as promoter-specific TFs [19, 53]. These findings agree with our expectation because ubiquitous DHSs largely overlap with promoter regions (see S3 Fig).

### Predicted co-occurring TF pairs show a significant enrichment of experimentally-validated PPIs

**Fig 4:**
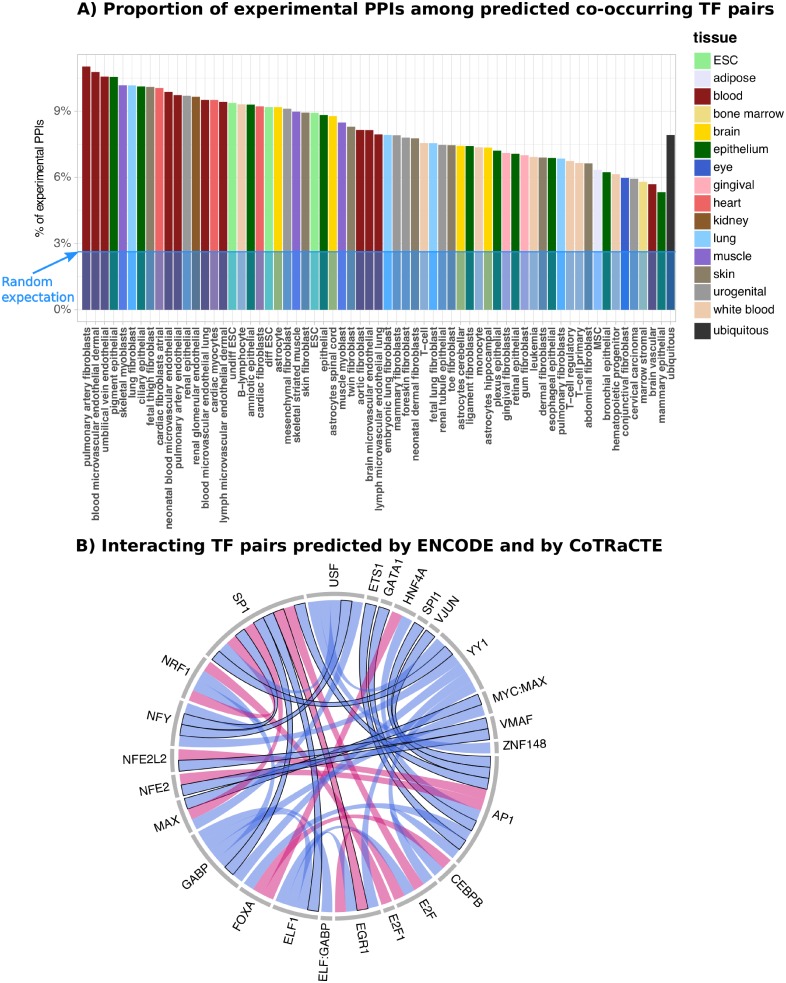
Comparison of predicted co-occurring TF pairs by CoTRaCTE with experimental data. A) Proportion of experimentally validated PPIs in the predicted co-occurring TF pairs by CoTRaCTE by cell type, including the predicted TF pairs on ubiq-DHSs. Blue level indicates the proportion of PPIs in a random set of TF pairs. B) Comparison of interacting TF pairs predicted by ENCODE and by CoTRaCTE. Blue cords denote the high confidence set of interacting TFs from ENCODE, TF pairs predicted also by CoTRaCTE are highlighted in red. Experimental PPIs are highlighted with black border lines.

To biologically verify our computationally predicted co-occurring TF pairs, we compared these with experimentally-validated direct protein-protein interactions (PPIs) between TFs. We compared our predicted co-occurring TF pairs with the atlas of TF-TF interactions inferred from mammalian two-hybrid assays [54] and from other forms of experimental evidence listed in PPI databases [55]. After individual TFs in both sets were assigned to each other, 169 TF pairs (7.1% out of 2376 predicted TF pairs) were found in both sets. This corresponds to a large enrichment relative to random expectation if there was no agreement between computational and experimental predictions (with an odds ratio of 2.0, and a corresponding *p*-value of *p* = 1.87 × 10^−14^, Fisher’s exact test). Among these validated TF pairs were included for example GATA and MEF2 in hematopoietic progenitor cells; GATA and SRF in primary T-cells; differentiated ESCs and in HPCs; E2F1 and NFKB in skin fibroblast; SMAD4 and FOS/JUN/AP1 in lung fibroblasts, ciliary epithelial and brain microvascular endothelial cells. Over all studied cell types, the highest proportion of experimentally validated PPIs was found among co-occurring TFs from pulmonary artery fibroblasts (11.0%) whereas the smallest proportion was found in mammary epithelial cells (5.3%). Among the co-occurring TF pairs within ubiq-DHS regions, 110 out of 1389 (7.9%) pairs were also found to be interacting proteins according to the PPI data (corresponding *p*-value = 1.8 × 10^−6^, Fisher’s exact test). The proportions of experimental PPIs among predicted co-occurring TF pairs for all cell types are shown in Fig 4. Notably, these proportions are much larger than the proportion (2, 7%) which would be expected by a random selection of the same number of TF pairs.

### Predicted co-occurring TF pairs are significantly enriched for TF cooperative pairs identified by ChIP-seq experiments

Next, we compared our computational predictions with highly precise experimental predictions of TF cooperativity derived from the chromatin immunoprecipitation technique coupled with high-throughput sequencing (ChIP-seq). The largest available experimental mapping of TF binding regions in human cell lines using the ChIP-seq technique was generated by the ENCODE Project [13]. In an accompanying study, [8] analyzed all 457 ChIP-seq data sets for 87 sequence-specific human TFs in 72 cell lines to determine binding cooperativity for different pairs of TFs. The authors identified peak regions bound by a primary TF and then conducted an overrepresentation analysis for secondary motifs associated with additional TFs. In this way, they identified a total of 155 putatively co-binding TF pairs among 69 investigated TFs. Of the 155 TF pairs identified by [8], 120 were found to be represented in our dataset of putatively co-occurring TF pairs after assignment of the TF motifs across datasets. Of these 120 pairs, 10 were found by CoTRaCTE within CTS-DHS regions (odds ratio = 2.4, *p*-value = 0.02, Fisher’s exact test). For example, IRF4 and PAX5 cooperativity was previously identified in the ChIP-seq experiment in lymphoblastoid cell line (GM12878) and our method predicted this TF pair as co-occurring in B-lymphocytes, T-cell, fibroblasts and in astrocytes. Notably, the interaction of these TFs has been previously described as relevant to the innate immune response [56]. A further example is provided by the known interaction partners STAT1 and CEBPB which were not only detected in the HeLa ChIP-seq experiment but also predicted with our method as co-occurring in diverse fibroblasts. Furthermore, the ChIP-seq read counts of CEBPB in the peak regions of STAT1 are correlated (with *r* > 0.3) in lymphoblastoid (GM12878) and in leukemia (K562) cell lines [8]. All TF pairs predicted both computationally by CoTRaCTE and experimentally by the ChIP-seq based method of [8] are listed in Table 2, including the cell type where the TF pair was predicted to co-occur as well as evidence from the literature for known interactions between TF pairs. Among TF pairs identified by CoTRaCTE but not identified by the ChIP-seq method, 19 TF pairs were found as experimental PPIs in BIOGRID database. All the pairs not identified by ChIP-seq including additional information are listed in S4 File.

**Table 2:** Co-binding TF pairs derived by ChIP-seq experiments found predicted as co-occurring TF pairs with our method, including the predicted cell type and literature citations.

TF pair	cell type	literature evidence
MYC:YY1	fibroblast and epithelial cells	direct PPI [55]
TBP:YY1	esophaegal epithelial cells	none
STAT1:CEBPB	fibroblast	direct PPI [55]
HNF4:ESRRA	HeLa cells, skeletal muscle	direct PPI [55]
HNF4A:TCF12	muscle myoblast, microvascular endothelial dermal cells	none
HNF4A:SP1	fibroblast	direct PPI [55]
IRF4:PAX5	B-lymphocyte, T-cell, fibroblast, astrocytes	predicted PPI [56]
IRF4:MEF2A	primary T-cell	none
TAL1:STAT3	fibroblast	none
RXRA:TCF7L2	differentiated ESCs	none
E2F4:NRF1	ubiquitous	none
JUN:FOXA	ubiquitous	none

Further, we compared our computational predictions with the high confidence set representing TF-TF interactions from ENCODE [8] which is based not only on the ChIP-seq experiments but includes information from other ChIP-seq datasets with an analysis of preferred binding arrangements of the heterotypic TFs. Similarly to the previous comparison, we found a significant overlap between co-occurring TF pairs predicted by CoTRaCTE and TF pairs in the high confidence set of interacting TFs(odds ratio = 7.2, *p*-value = 3.9 ⋅ 10^−5^). The high confidence set of interacting TFs from ENCODE is visualized in Fig 4B, TF pairs predicted also by CoTRaCTE are highlighted in red. Notably, three TF pairs SP1:EGR1, SP1:E2F1 and AP1:NFE2L2 were predicted by both, CoTRaCTE and ENCODE and are known interacting proteins in the BIOGRID database [55]).

### Predicted co-occurring TF pairs are found among top-predicted TF-TF dimers

As a positive control, we next compared our predicted co-occurring TF pairs with a dataset generated by a computational method for predicting cooperative cell-type specific dimerization of TFs on the DNA molecule [17]. This dataset considered the occurrence of more than 450000 TF motif pairs in cell-type specific DHSs in 78 cell types while accounting for the orientation and spacing of the two motifs. Based on ∼1.4 billion tests for enrichment of TF motif pairs with specific orientation and spacing, 603 highly significant cell-type specific TF-TF dimers were predicted.

There is a relatively low agreement between the co-occurring TF motifs predicted by our method with the set of TF-TF dimers predicted by [17] with only 44 out of 603 TF pairs (7.3%, *p*-value = 0.37, Fisher’s exact test) identified by both methods. Nevertheless, 5 of the top-10 most significant predicted TF-dimers (E-box dimer, OCT-SOX heterodimer, IRF homotypic dimer, EBF1 dimer, FOXA1:AR dimer), were also predicted by our method. Notably, since 3 of the top-10 predicted TF dimers are homodimers (which can not be predicted using our method which only considers cooperativity between two distinct TFs) this leaves only 2 out of the top-10 predictions which remain undetected by our method. The dimerization of all top-10 predicted TF dimers has been independently confirmed in other experimental studies, see S2 Table for summary.

### Predicted ESC-specific regulatory networks confirm previous findings

**Fig 5:**
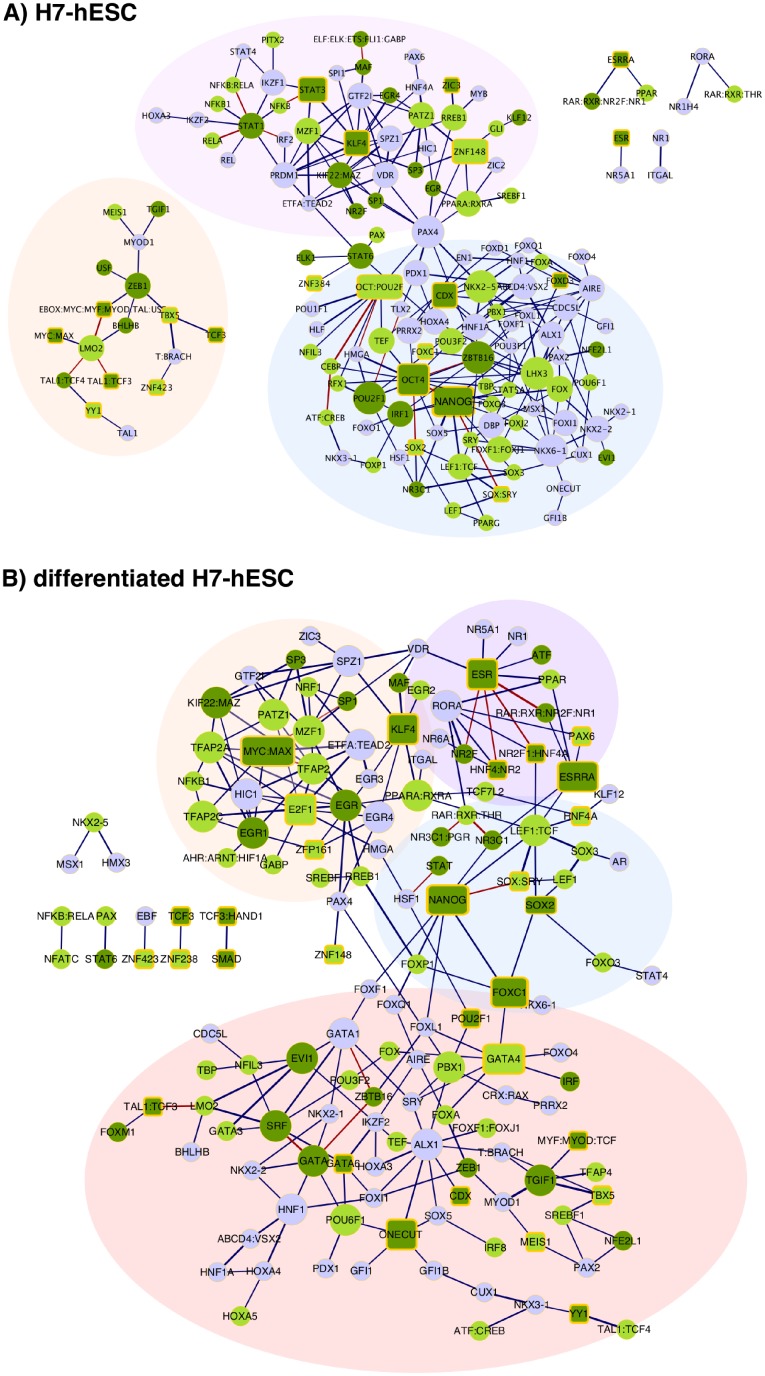
Network of predicted co-occurring TF pairs in undifferentiated A) and differentiated B) embryonic stem cells (H7-hESC cell line). A) Network of predicted co-occurring TFs in ESCs. Nodes in the network represent transcription factors, edges are drawn between co-occurring TF pairs predicted by coTRaCTE. Red edges are known protein-protein interactions which are also predicted by coTRaCTE. TFs expressed in the cell line are highlighted in green; darker tone indicates stronger evidence of expression in related cell types. Known regulators in ESCs are highlighted as rectangles with yellow border. Node size reflects the number of predicted co-occurring TF partners. Three subnetworks with distinct regulatory functions are highlighted: maintanance of pluripotency (blue); embryonic development (pink) and general functions (orange). B) Network of predicted co-occurring TFs in differentiated ESCs. Four subnetworks with distinct regulatory functions are highlighted: pluripotency (blue); early development of organs and tissues (red); mesoderm differentiation (purple) and general functions (orange).

Besides testing our method globally using 64 different cell types (see S3 File), we conducted a detailed local analysis of the TF networks in embryonic stem cells (ESCs), by contrasting undifferentiated H7-hESC cells and differentiated H7-hESC cells (see S1 Appendix and S10 Fig for the local analysis of hematopoietic progenitor cells and K562 cells). To investigate the occurrence of cell-type specific TFs, all TF pairs appearing in 30 or more cell lines were removed from the dataset and cell-type specific regulatory networks were constructed using all remaining significantly co-occurring TF pairs. The predicted regulatory networks are shown in Fig 5 where nodes correspond to TFs and pairs of TFs predicted to co-occur are connected with edges.

The predicted ESC networks consist of 216 and 234 predicted co-occurring TF pairs for differentiated H7-hESC and undifferentiated H7-hESC cells, respectively. These pairs respectively comprise 127 and 147 distinct TFs of which the majority (67 and 76%) has been shown to be expressed in ESCs ([46]; green nodes in Fig 5). Moreover, approximately 20% of the TFs are known to function in pluripotency or early development (rectangle nodes with yellow border in Fig 5). We conclude that the known expression and function of these TFs may provide independent evidence for their potential activity and co-regulation in ESCs.

The main regulators in the predicted network in undifferentiated ESCs are OCT4, NANOG, SOX2, POU2F1, LHX3, ZBTB16 and PAX4 (see Fig 5A). Accordingly, the most important human pluripotent factors OCT4, NANOG and SOX2 [26] have the highest number of predicted TF partners. Further, other pluripotent factors such as MYC and KLF4 and the majority of known early developmental regulators such as STAT3, FOXD3, ESRR, TCF3, zinc finger proteins and YY1 [26, 57] are also present in our predicted network. Previous studies of ESCs have shown that the main pluripotent factors OCT4, NANOG and SOX2 bind in complexes to the regulatory regions of their target genes [57]. This experimental finding is in agreement with our predictions as illustrated by the TF pairs OCT4:NANOG, OCT4:SOX2, NANOG:SOX/SRY which we predicted as co-occurring in the ESC-specific DHS regions. Further, our predicted ESC networks contain other experimentally verified PPIs including NANOG:TCF/LIF1, CEBP:OCT/POU2F, STAT3:NFKB and OCT/POU2F:TBP (red edges in Fig 5).

### Predicted ESC-specific regulatory network contains three subnetworks with distinct regulatory functions

Our predicted undifferentiated ESC-specific network consists of three separated subnetworks connected only via PAX4 and STAT6. The delineation of these subnetworks suggests that they perform different functions during ESC regulation. One subnetwork includes the main pluripotent factors OCT4, NANOG, SOX together with CDX, FOXD3, ZBTB16, LHX3, as well as other FOX genes and the NK-Homeoboxes (blue subnetwork in Fig 5A). These TFs are involved in regulation of development decisions and chromatin remodeling and in the maintenance of the pluripotency [25, 26]. For example, the association of OCT4 and ZBTB16 was shown in the transcriptional network derived by [58].

The second subnetwork (highlighted in pink in Fig 5A) includes regulators such as KLF4, STAT3, ZIC3 and ZNF148 which are all known targets of the pluripotent factors OCT4, NANOG and SOX2 and regulators of early embryonic development [19, 25]. Our findings suggest that this subnetwork might be activated by the pluripotent factors and might carry out a distinct function from that of the first subnetwork. Correspondingly, a distinct regulatory mechanism of KLF4 compared to the subnetwork consisting of OCT4, SOX2 and NANOG has been previously suggested [25].

Finally, we identified a third subnetwork including known ESC-regulators such as MYC/MAX, TCF3, TBX5 and YY1 (orange subnetwork in Fig 5A). This subnetwork functions mainly in the regulation of stem cell differentiation and embryonic organ development [45]. Our identification of this subnetwork is supported by three types of independent evidence. First, [12] found that the the core pluripotent factors OCT4, SOX2 and NANOG co-regulate their target genes in the absence of MYC consistent with our prediction that MYC is localised in a separate subnetwork. Second, the findings of [57] that KLF4 and ESRR co-occurr more frequently with OCT4 than with MYC confirms the separation of this subnetwork. Third, YY1 has also been shown to be an active component of the MYC transcription network in ESCs [59] which is in agreement with our results.

### The predicted regulatory networks of differentiated and undifferentiated ESCs show substantial differences

For comparison with undifferentiated cells, we investigated the predicted regulatory network of co-occurring TFs in the differentiated H7-hESC cell line. This predicted network shows clear differences from that of undifferentiated ESCs (see Fig 5B) and can be divided into four subnetworks. The first subnetwork (highlighted in blue in Fig 5B) consists of the pluripotent factors SOX2, NANOG, FOXC1 and is connected to the second subnetwork (highlighted in red in Fig 5A) dominated by the GATA proteins which are known for their important roles in transducing nuclear events that regulate cellular differentiation and embryonic morphogenesis [60]. Following an analysis conducted with GeneMANIA [45], we observe a clustering of GATA proteins with several TFs involved in the development of organs and tissues such as endocrine system (ONECUT, NKX2-1,NKX2-2,HNF1B), muscles (SRF, POU6F1) and hematopoiesis (TAL1, EVI1, ZBTB16). The third subnetwork (highlighted in orange in Fig 5B) is also connected with the pluripotent factors and is dominated by the transcription factor ESR (which possesses similar motifs to those of ESRRA and ESRRB). ESR is the most important target of NANOG and serves to maintain cell pluripotency [61, 62]. In our predicted regulatory network, ESR clusters with the HNF4 and NR2F transcription factors which are important regulators of mesoderm differentiation. The fourth subnetwork (highlighted in blue in Fig 5B) is MYC-centered and includes the transcription factors E2F1, KLF4 as well as zinc finger proteins and the general regulators TFAP2 and SP1. This finding agrees with the observation of MYC-centric complexes consisting of E2F1, the zinc finger protein ZFX and CTCF in a previous ChIP-seq study [57]. In conclusion, the predicted transcriptional network in differentiated ESCs includes more cases of pluripotent TFs co-occurring with TFs involved in early cell differentiation than the predicted transcriptional network in undifferentiated ESCs.

### GATA1 partners with different co-factors in different cell types

**Fig 6:**
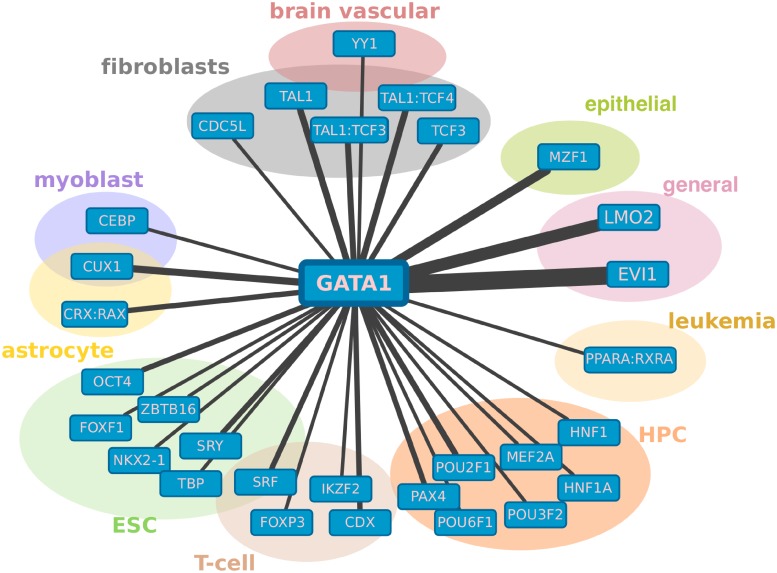
Predicted co-factors of GATA1 in different cell types. Nodes in the network represent transcription factors, edges are drawn between the co-occurring TF pairs predicted by coTRaCTE, the width of each edge corresponds to number of cell types for which the TF co-occurrence was predicted. Coloured groups indicate different cell types for which the TF co-occurrence was predicted.

Another insight into the transcriptional regulatory mechanism is provided by coTRaCTE. Specifically, it can be used to investigate the predicted co-factors of a specific TF of interest in various cell types. As a proof-of-principle, we demonstrate this type of TF-centric analysis using GATA1, see Fig 6. GATA1 is a protein which plays an important role in erythroid development [42, 43] but is also expressed in many other cell types [47] suggesting that it regulates diverse functions in different cell types. Using our approach, we identified several TFs that co-occur with GATA1 in a cell-type specific manner. For example, GATA1 partners with HNF1 in hematopoietic progenitor cells; with PPARA:RXRA in leukemia; with SRF, CDX and FOXP3 in primary T-cells; with SRY, NKX2-1, FOXF1, OCT4 and ZBTB16 in differentiated ESCs and with TAL1:TCF3 motif co-occurring in various fibroblasts. Strikingly, EVI1 was identified as a co-factor of GATA in 19 cell types, indicating that it serves as a more general partner of GATA1. EVI1 is thought to be involved in hematopoiesis, development and cell differentiation as part of the MECOM complex [42, 43] and is expressed in a large number of tissues [47]. This evidence suggests that EVI1 (MECOMB) is a general transcriptional co-factor of GATA1.

## Discussion

Here, we present coTRaCTE, a statistical method for detecting putatively cooperative TF pairs co-occurring in a cell-type specific manner within accessible chromatin regions. Our approach incorporates two novel refinements which guarantee highly specific predictions of TF cooperativity and which address the limitations of previous methods.

First, coTRaCTE distinguishes cell-type specific DNase hypersensitive sites (CTS-DHSs) from ubiquitous DNase hypersensitive sites (ubiq-DHSs) using 90 DNase-seq experiments and employing a *t*-statistic-based measure. This statistical method, which was previously applied to another data set [29], provides a ranking of genomic sites that are consistently DNase hypersensitive in a given cell type, relative to an average profile of all studied cell types. By analysing 64 different cell types in this way, we predict not only chromatin regions that are open in a cell-type specific manner (i.e. cell-type specific enhancers) but also chromatin regions that are open ubiquitously among all cells.

Second, coTRaCTE determines the cooperativity of TF pairs by contrasting their co-occurrence in the cell-type specific enhancers and ubiquitously open regions. Using the TRANSFAC database of TF binding motifs and the predicted TF binding affinity for both types of region, we determine bound and unbound DHSs for each individual TF. Then, for each pair of TFs, we quantify the overlap between the cell-type specific regions bound by the TF pair using a Fisher’s exact test. We then quantify the overlap between the ubiquitously open regions bound by the TF pair using a second Fisher’s exact test. Cell-type specific regions and ubiquitous regions bound by both TFs are then compared using the log-ratio of *p*-values from the two Fisher’s exact tests. Notably, by using the ubiquitous regions as background coTRaCTE can detect cooperative TF pairs that are cell-type specific.

Using this approach we predicted 2359 TF pairs as co-occurring in the cell-type specific enhancers of 64 cell types. The large majority (70%) of these pairs are either highly specific for a single cell type or show a large degree of overlap in their predicted TF pairs among related cell types originating from the same tissue. Conversely, we identified 158 TF pairs common to at least 30 out of 64 cell types. According to our observations, these TF pairs are more likely to co-occur within cell-type specific enhancers than within ubiquitously open chromatin regions regardless of the cell type considered. This finding agrees with a recent study of accessible chromatin regions [50] which described partial sequence similarity among cell-type specific DHSs. Interestingly, the set of TF pairs found to preferentially co-occurre on ubiquitously open chromatin in contrast to enhancers specific to a given cell-type is almost invariant regardless of the cell-type considered. This finding confirms our expectation that cell-type specific transcriptional regulation takes place mainly within cell-type specific enhancers, whereas general regulation takes place mainly within ubiquitously open chromatin regions. Importantly, the enrichment of individual TFs within cell-type specific enhancers does not show such a clear cell-type specific pattern than that seen for TF pairs. Thus, the cooperative TF pairs contribute additional information on top of the single TF overrepresentation analysis. This result confirms previous findings that regulatory decisions are usually governed by a specific combination of TFs that act cooperatively rather than individually [2, 63].

To assess the validity of our predictions, we derived cell-type specific regulatory networks from the predicted TF pairs in each particular cell type and investigated TFs present in these networks. In general, more than 75% of TFs in the networks are expressed in the particular cell type as measured by an independent study using RNA-seq experiments [46]. Moreover, roughly one quarter of TFs in the cell-type specific networks are known regulators in the particular cell type. Notably, all TFs in the predicted networks were selected only by their high *L*_*ct*_ score without any knowledge of their possible function or expression in the corresponding cell type. This fact underlines the plausibility of our predictions.

To validate our results systematically, we compared the predicted co-occurring TF pairs with (i) large-scale experimental databases of PPIs [54, 55], (ii) predictions derived from an analysis of ChIP-seq experiments [8] and (iii) a statistical prediction of TF-dimerization [17]. The experimentally-determined PPIs and relationships between TFs derived from ChIP-seq are significantly enriched among our predicted set of cell-type specific co-occurring TFs. When comparing coTRaCTE predictions with the experimentally derived database of direct PPIs, it is important to consider the sensitivity (true positive rate) and the false discovery rate (FDR) of the experimental method, estimated by [54] to be 25% and 53%, respectively. For this reason, even if the coTRaCTE predictions showed optimal sensitivity, we could only expect a maximum of 25% of the computationally predicted TF pairs to be represented in the experimentally derived atlas. Similarly, even if the coTRaCTE predictions showed optimal specificity, we could only expect a maximum of 47% of the experimentally determined PPIs to be represented among the computationally predicted TF pairs. Furthermore, the differing results from these two methods are consistent with the fundamental methodological differences between coTRaCTE (which considers cooperative TF binding on the DNA molecule) and the experimental approaches to detect PPIs (which measure the general ability of two proteins to form a complex).

Interestingly, the agreement between the predicted TF-TF dimers [17] and our predicted set of co-occurring TFs is relatively low (7%). Nevertheless, out of the top seven most significant predicted TF-TF heterodimers having further supporting evidence from the literature, five were also represented in our set of predicted co-occurring TF pairs. This leaves only 2 of the top 7 predicted TF heterodimers undetected by our method. The relatively small concordance between the coTRaCTE set of predictions and the set of TF-TF dimer predictions might be explained by the differing rationales of both prediction methods. Our method is designed to predict pairs of TFs which co-occur preferentially in cell-type specific enhancers compared to ubiquitously open chromatin regions. In contrast, the predicted TF-TF dimer set consists of directly interacting TFs which bind as a dimer to regulatory DNA regions with a fixed spacing and orientation [17].

Apart from validating the coTRaCTE predictions globally across a range of cell types, we performed a detailed local analysis of the predicted TF networks in embryonic stem cells. As expected, we recovered the core pluripotent factors OCT4, NANOG and SOX2 as the dominant regulators in undifferentiated ESCs as well as recovering the known co-binding of these three TFs. However, we also observed the early developmental regulators such as KLF4, ESRR and MYC in the predicted networks, as well as several known direct protein interactions (e.g. NANOG:TCF/LIF1, CEBP:OCT4, STAT3:NFKB). The predicted network in undifferentiated ESCs is characterized by three subnetworks performing distinct regulatory functions. One subnetwork including OCT4, SOX2 and NANOG is responsible for the maintenance of the pluripotency. The second subnetwork consists of several regulators of early embryonic development such as KLF4, STAT3, ZIC3 and ZNF148, which are known to be direct targets of the core pluripotent factors. The third subnetwork contains MYC/MAX proteins and is completely isolated from the other subnetworks, consistent with several previous studies [12, 57, 59]. In contrast, the predicted regulatory network in differentiated ESCs includes transcriptional regulators with a higher degree of cell-specificity such as LEF1/TCF, GATA4, EGR and TFAP2. In the transcriptional network for differentiated ESCs, we identified four smaller subnetworks which perform the following functions: (1) pluripotency determined by a subnetwork consisting of SOX and NANOG; (2) early development of organs and tissues; (3) mesoderm differentiation and (4) a subnetwork carrying out more general functions. These results suggest that several cell-type specific TFs are highly active after only a few days of ESC differentiation and that this can drive cell differentiation along a developmental trajectory to the determined cell type. In addition to predicting regulatory networks for a particular cell type of interest, coTRaCTE provides information about the co-regulators of a selected TF in various cell types. For example, for GATA1, we found several cell-type specific co-regulators such as HNF1 in hematopoietic progenitor cells, PPARA:RXRA in leukemia and a general co-regulator EVI1 found to cooperate with GATA1 in more than 15 cell types.

Overall, the validation of our predicted co-occurring TF pairs and further analysis of cell-type specific networks confirms that these predictions include a significant proportion of TFs independently identified as either co-occurring or directly interacting. Moreover, the large majority of regulators observed in the transcriptional network specific to a given cell type are actually expressed in the corresponding cell type. In addition, roughly one quarter of these regulators are known to function in the corresponding cell type. Thus, our findings are not only verified by previously reported observations but also reveal novel potential TF co-occupancies that can be validated by further experimentation. Despite these advantages of coTRaCTE, the method also has some limitations. First, it is insensitive to homodimers because we can recognize an interaction only when two different binding sites are bound. Another question that might arise concerns the detection of competitively binding TFs. coTRaCTE clearly is not designed to detect this since our approach does not involve a physical interaction between the competing factors. It would, nevertheless, appear feasible to design an alternative analysis of the cell-type specific DHSs aiming at the delineation of competing factors, e.g., by including cell-type specific expression data.

In summary, our predicted co-occurring TFs provide further insight into cell-type specific combinatorial regulation by transcription factors. We recommend coTRaCTE as a powerful tool for the generation of statistically-rigorous predictions of cooperativity between TF pairs thus accelerating the elucidation of gene regulatory networks not just in human but in any species for which chromatin accessibility data is available.

## Supporting information

S1 Appendix Supporting information. (PDF)

S1 Fig Dependency of the significance in Fisher’s exact test on threshold selection for 477 TF motifs in three different cell lines. Dependency of the significance in Fisher’s exact test on threshold selection for 477 TF motifs in (a) leukemia, (b) embryonic stem cells (ESCs) and (c) B-lymphocytes. The significance is represented as − log_10_ *p*-value on the vertical axis. 11 combinations of thresholds *k* (defining the top-ranked DHSs) and *t* (defining the number of cell-type specific and ubiquitous DHSs) is depicted on the horizontal axis. Top-20 enriched TF motifs selected with two extreme values of *k* and *t* are highlighted in red and blue, respectively.
(EPS)

S2 Fig Consistency of the most significant TF pairs on CTS-DHSs for different combinations of parameters. Consistency of the most significant TF pairs on CTS-DHSs for different combinations of parameters in a) embryonic stem cell (ESC) and b) B-lymphocyte. The matrix entries denote the number of identical TF pairs with the highest *L*_*l*_ score for 11 combinations of thresholds *k*_1_ = *k*_2_ (the first number) and of threshold *t* (second number).
(EPS)

S3 Fig Genomic distribution of the top 5000 CTS-DHSs and of the top 5000 ubiquitous DHSs. Genomic distribution of the 5000 most cell-type specific DNase hypersensitive sites in 64 cell types and of the top 5000 ubiquitous DNase hypersensitive sites sorted by the overlap with promoter regions.
(EPS)

S4 Fig Overrepresented transcription factors over 64 cell types. Each cell in the matrix indicates the significance of the association between the cell type and the corresponding TF. TFs overrepresented in the majority of cell types are highlighted in red. Cell type-specific TFs are marked with boxes of color corresponding to the tissue.
(EPS)

S5 Fig Network of highly frequent TF pairs predicted in at least 30 out of 64 cell types. Nodes in the network represent transcription factors, edges are drawn between the co-occurring TF pairs predicted by CoTRaCTE. Red edges are known protein-protein interactions.
(EPS)

S6 Fig Network of co-occurring TF pairs in ubiquitous DHSs. Nodes in the network represent transcription factors, edges are drawn between the co-occurring TF pairs predicted by CoTRaCTE. Red edges are known protein-protein interactions. Known promoter-specific regulators are highlighted as rectangles with red border; green nodes are TFs indicated as overrepresented on promoter sequences in [4].
(EPS)

S7 Fig Comparison of co-occurring TF pairs in undifferentiated and differentiated embryonic stem cells. Comparison of predicted regulators in undifferentiated and differentiated embryonic stem cells. For each TF, the barplot shows the number of distinct co-occurring partners in undifferentiated ESCs (red) and in differentiated ESCs (blue) and the number of shared co-occurring partners on both cell lines (black). The left column shows the absolute numbers, the right column shows the proportions.
(EPS)

S8 Fig Heatmap of overlapping predicted co-occurring TF pairs on ubiq-DHSs over 64 cell types. Each cell depicts the number of TF pairs shared between the corresponding pair of cell types.
(EPS)

S9 Fig Boxplots showing the distributions of GC-content in the ubiq-DHSs and CTS-DHSs by cell type. Each boxplot shows the GC-content distribution of the 5000 most cell-type specific and most ubiquitous DHSs, respectively. The boxes of each cell type are coloured by the corresponding tissue. Blue line depicts the average GC content of the human genome (hg19) which is 40.9%.
(EPS)

S10 Fig Network of predicted co-occurring TF pairs in hematopoietic progenitor cells and leukemia. A) Network of predicted co-occurring TFs in hematopoietic progenitor cells. Nodes in the network represent transcription factors, edges are drawn between co-occurring TF pairs predicted by coTRaCTE. Red edges are known protein-protein interactions which are also predicted by coTRaCTE. TFs expressed in the cell line are highlighted in green; darker tone indicates stronger evidence of expression in related cell types. Known regulators in hematopoiesis are highlighted as rectangles with yellow border. Node size reflects the number of predicted co-occurring TF partners. B) Network of predicted co-occurring TFs in leukemia.
(EPS)

S1 Table Most significant cell-type specific TFs in various cell types. TFs in bold are known transcription regulators in the corresponding cell type.
(PDF)

S2 Table Top-10 predicted TF-TF dimers by Jankowski *et al*. [17] compared to predictions of coTRaCTE. Predicted TF-TF dimers by [17] with the predicted cell type (first two columns); predicted co-occurring TF pairs by coTRaCTE including the predicted cell type (third and fourth column) and literature evidence (fifth column).
(PDF)

S1 File DNase-seq data from ENCODE project used in the analysis. (XLSX)

S2 File List of 554 TF motifs and corresponding TF groups. List of all 554 TF motifs from TRANSFAC database used for the analysis and their corresponding TF names(groups). Table includes an alternative factor name and further information from TRANSFAC about the availability and quality of the position weight matrix (PWM).
(CSV)

S3 File Predicted co-occurring TF pairs in 64 cell types. Predicted co-occurring TF pairs in separate files for each cell type. The columns show the following: TF 1 name, TF 2 name, − log_10_(*p*-value_CTS_), − log_10_(*p*-value_ubiq_), *L*_*l*_ score, frequency of the TF pair in other cell types, known PPI (1 = yes, 0 = no), motif similarity MOSTA.
(ZIP)

S4 File Predicted co-occurring TF pairs by CoTRaCTE and by ENCODE. Predicted co-occurring TF pairs that are comparable with the ENCODE predictions. The columns show the following: TF 1 name, TF 2 name, prediction by ENCODE, known PPI (1 = yes, 0 = no), Ensembl ID1, Ensembl ID2 and other experimental evidence.
(XLSX)

## References

[1] Maston Glenn A. and Evans Sara K. and Green Michael R.(2006). Transcriptional Regulatory Elements in the Human Genome. *Annual Review of Genomics and Human Genetics*, 7, 29–59. 10.1146/annurev.genom.7.080505.115623 16719718

[2] Zhu Zhou and Shendure Jay and Church George M. (2005). Discovering functional transcription-factor combinations in the human cell cycle. *Genome Research*, 15, 848–855. 10.1101/gr.3394405 15930495 PMC1142475

[3] Hu Z. and Gallo S. (2010). Identification of interacting transcription factors regulating tissue gene expression in human. *BMC Genomics*, 11, 49+ 10.1186/1471-2164-11-49 20085649 PMC2822763

[4] Myšičková A. and Vingron M. (2012). Detection of interacting transcription factors in human tissues using predicted DNA binding affinity. *BMC Genomics*, 13 Suppl 1, S2+ 10.1186/1471-2164-13-S1-S2 22369666 PMC3583127

[5] Vandenbon A., Kumagai Y., Akira S., and Standley D. (2012). A novel unbiased measure for motif co-occurrence predicts combinatorial regulation of transcription. *BMC Genomics*, 13, S11+ 10.1186/1471-2164-13-S7-S11 23282148 PMC3521209

[6] Ha N., Polychronidou M., and Lohmann I. (2012). COPS: Detecting Co-Occurrence and Spatial Arrangement of Transcription Factor Binding Motifs in Genome-Wide Datasets. *PLoS ONE*, 7, e52055+ 10.1371/journal.pone.0052055 23272209 PMC3525548

[7] Meckbach C., Tacke R., Hua X., Waack S., Wingender E., and Gültas M. (2015). PC-TraFF: identification of potentially collaborating transcription factors using pointwise mutual information. *BMC bioinformatics*, 16 10.1186/s12859-015-0827-2 26627005 PMC4667426

[8] Wang J., Zhuang J., Iyer S., Lin X. Y., Whitfield T. W., Greven M. C., Pierce B. G., Dong X., Kundaje A., Cheng Y., Rando O. J., Birney E., Myers R. M., Noble W. S., Snyder M., and Weng Z. (2012). Sequence features and chromatin structure around the genomic regions bound by 119 human transcription factors. *Genome Res*., 22, 1798–1812. 10.1101/gr.139105.112 22955990 PMC3431495

[9] Gerstein M. B., Kundaje A., Hariharan M., Landt S. G., Yan K.-K., Cheng C., Mu X. J., Khurana E., Rozowsky J., Alexander R., Min R., Alves P., Abyzov A., Addleman N., Bhardwaj N., Boyle A. P., Cayting P., Charos A., Chen D. Z., Cheng Y., Clarke D., Eastman C., Euskirchen G., Frietze S., Fu Y., Gertz J., Grubert F., Harmanci A., Jain P., Kasowski M., Lacroute P., Leng J., Lian J., Monahan H., O/’Geen H., Ouyang Z., Partridge E. C., Patacsil D., Pauli F., Raha D., Ramirez L., Reddy T. E., Reed B., Shi M., Slifer T., Wang J., Wu L., Yang X., Yip K. Y., Zilberman-Schapira G., Batzoglou S., Sidow A., Farnham P. J., Myers R. M., Weissman S. M., and Snyder M. (2012). Architecture of the human regulatory network derived from ENCODE data. *Nature*, 489, 91–100. 10.1038/nature11245 22955619 PMC4154057

[10] Whitington T., Frith M. C., Johnson J., and Bailey T. L. (2011). Inferring transcription factor complexes from ChIP-seq data. *Nucleic Acids Res*., 39, e98 10.1093/nar/gkr341 21602262 PMC3159476

[11] Oh Y. M. M., Kim J. K. K., Choi S., and Yoo J.-Y. Y. (2012). Identification of co-occurring transcription factor binding sites from DNA sequence using clustered position weight matrices. *Nucleic Acids Res*., 40, e38 10.1093/nar/gkr1252 22187154 PMC3300004

[12] Lee Yuju and Zhou Qing (2013). Co-regulation in embryonic stem cells via context-dependent binding of transcription factors. *Bioinformatics (Oxford, England)*, 29, 2162–2168. 10.1093/bioinformatics/btt365 23793746

[13] The ENCODE Project consortium (2012). An integrated encyclopedia of DNA elements in the human genome. *Nature*, 489, 57–74. 10.1038/nature11247 22955616 PMC3439153

[14] Park S.-J. J., Umemoto T., Saito-Adachi M., Shiratsuchi Y., Yamato M., and Nakai K. (2014). Computational promoter modeling identifies the modes of transcriptional regulation in hematopoietic stem cells. *PloS ONE*, 9. 10.1371/journal.pone.0093853 PMC3977923 24710559

[15] Blatti C., Kazemian M., Wolfe S., Brodsky M., and Sinha S. (2015). Integrating motif, DNA accessibility and gene expression data to build regulatory maps in an organism. *Nucleic Acids Res*., 43, 3998–4012. 10.1093/nar/gkv195 25791631 PMC4417154

[16] Kazemian M., Pham H., Wolfe S. A., Brodsky M. H., and Sinha S. (2013). Widespread evidence of cooperative DNA binding by transcription factors in Drosophila development. *Nucleic Acids Res*., 41, 8237–8252. 10.1093/nar/gkt598 23847101 PMC3783179

[17] Jankowski A., Szczurek E., Jauch R., Tiuryn J., and Prabhakar S. (2013). Comprehensive prediction in 78 human cell lines reveals rigidity and compactness of transcription factor dimers. *Genome Res*., 23, 1307–1318. 10.1101/gr.154922.113 23554463 PMC3730104

[18] Jankowski A., Prabhakar S., and Tiuryn J. (2014). TACO: a general-purpose tool for predicting cell-type-specific transcription factor dimers. *BMC Genomics*, 15, 208+ 10.1186/1471-2164-15-208 24640962 PMC4004051

[19] Neph S., Vierstra J., Stergachis A. B., Reynolds A. P., Haugen E., Vernot B., Thurman R. E., John S., Sandstrom R., Johnson A. K., Maurano M. T., Humbert R., Rynes E., Wang H., Vong S., Lee K., Bates D., Diegel M., Roach V., Dunn D., Neri J., Schafer A., Hansen R. S., Kutyavin T., Giste E., Weaver M., Canfield T., Sabo P., Zhang M., Balasundaram G., Byron R., MacCoss M. J., Akey J. M., Bender M. A., Groudine M., Kaul R., and Stamatoyannopoulos J. A. (2012). An expansive human regulatory lexicon encoded in transcription factor footprints. *Nature*, 489, 83–90. 10.1038/nature11212 22955618 PMC3736582

[20] Xi H., Shulha H. P., Lin J. M., Vales T. R., Fu Y., Bodine D. M., McKay R. D., Chenoweth J. G., Tesar P. J., Furey T. S., Ren B., Weng Z., and Crawford G. E. (2007). Identification and characterization of cell type-specific and ubiquitous chromatin regulatory structures in the human genome. *PLoS Genetics*, 3, e136+ 10.1371/journal.pgen.0030136 17708682 PMC1950163

[21] Heintzman Nathaniel D and Stuart Rhona K and Hon Gary and Fu Yutao and Ching Christina W and Hawkins R David and Barrera Leah O and Van Calcar Sara and Qu Chunxu and Ching Keith A and Wang Wei and Weng Zhiping and Green Roland D and Crawford Gregory E and Ren Bing (2007). Distinct and predictive chromatin signatures of transcriptional promoters and enhancers in the human genome. *Nature Genetics*, 39, 311–318. 10.1038/ng1966 17277777

[22] Bulger Michael and Groudine Mark (2010). Enhancers: The abundance and function of regulatory sequences beyond promoters. *Developmental Biology*, 339, 250–257. 10.1016/j.ydbio.2009.11.035 20025863 PMC3060611

[23] Thurman R. E., Rynes E., Humbert R., Vierstra J., Maurano M. T., Haugen E., Sheffield N. C., Stergachis A. B., Wang H., Vernot B., Garg K., John S., Sandstrom R., Bates D., Boatman L., Canfield T. K., Diegel M., Dunn D., Ebersol A. K., Frum T., Giste E., Johnson A. K., Johnson E. M., Kutyavin T., Lajoie B., Lee B.-K. K., Lee K., London D., Lotakis D., Neph S., Neri F., Nguyen E. D., Qu H., Reynolds A. P., Roach V., Safi A., Sanchez M. E., Sanyal A., Shafer A., Simon J. M., Song L., Vong S., Weaver M., Yan Y., Zhang Z., Zhang Z., Lenhard B., Tewari M., Dorschner M. O., Hansen R. S., Navas P. A., Stamatoyannopoulos G., Iyer V. R., Lieb J. D., Sunyaev S. R., Akey J. M., Sabo P. J., Kaul R., Furey T. S., Dekker J., Crawford G. E., and Stamatoyannopoulos J. A. (2012). The accessible chromatin landscape of the human genome. *Nature*, 489(7414), 75–82. 10.1038/nature11232 22955617 PMC3721348

[24] Wang J., Rao S., Chu J., Shen X., Levasseur D. N., Theunissen T. W., and Orkin S. H. (2006). A protein interaction network for pluripotency of embryonic stem cells. *Nature*, 444, 364–368. 10.1038/nature05284 17093407

[25] Kim J., Chu J., Shen X., Wang J., and Orkin S. H. (2008). An Extended Transcriptional Network for Pluripotency of Embryonic Stem Cells. *Cell*, 132, 1049–1061. 10.1016/j.cell.2008.02.039 18358816 PMC3837340

[26] Chen X., Vega V. B., and Ng H. H. (2008b). Transcriptional Regulatory Networks in Embryonic Stem Cells. *Cold Spring Harb. Sym*., 73, 203–209. 10.1101/sqb.2008.73.026 19022762

[27] Parfitt D.-E. and Shen M. M. (2014). From blastocyst to gastrula: gene regulatory networks of embryonic stem cells and early mouse embryogenesis. *Philos. T. Roy Soc. B*., 369, 20130542+ 10.1098/rstb.2013.0542 PMC4216465 25349451

[28] Boyle A. P., Davis S., Shulha H. P., Meltzer P., Margulies E. H., Weng Z., Furey T. S., and Crawford G. E. (2008). High-resolution mapping and characterization of open chromatin across the genome. *Cell*, 132, 311–322. 10.1016/j.cell.2007.12.014 18243105 PMC2669738

[29] Ibn-Salem J., Koehler S., Love M. I., Chung H.-R., Huang N., Hurles M. E., Haendel M., Washington N. L., Smedley D., Mungall C. J., Lewis S. E., Ott C.-E., Bauer S., Schofield P. N., Mundlos S., Spielmann M., and Robinson P. N. (2014). Deletions of chromosomal regulatory boundaries are associated with congenital disease. *Genome Biol*., 15, 423+ 10.1186/s13059-014-0423-1 25315429 PMC4180961

[30] Quinlan Aaron R. and Hall Ira M. (2010). BEDTools: a flexible suite of utilities for comparing genomic features. *Bioinformatics (Oxford, England)*, 26, 841–842. 10.1093/bioinformatics/btq033 PMC2832824 20110278

[31] Roider H. G., Kanhere A., Manke T., and Vingron M. (2007). Predicting transcription factor affinities to DNA from a biophysical model. *Bioinformatics*, 23, 134–141. 10.1093/bioinformatics/btl565 17098775

[32] Kläning E., Christensen B., Bajic G., Hoffmann S. V., Jones N. C., Callesen M. M., Andersen G. R., Sørensen E. S., and Vorup-Jensen T. (2015). Multiple low-affinity interactions support binding of human osteopontin to integrin *α*X*β*2. *Biochimica et biophysica acta*, 1854(8), 930–938. 10.1016/j.bbapap.2015.03.008 25839998

[33] Crocker J., Abe N., Rinaldi L., McGregor A. P., Frankel N., Wang S., Alsawadi A., Valenti P., Plaza S., Payre F., Mann R. S., and Stern D. L. (2015). Low affinity binding site clusters confer hox specificity and regulatory robustness. *Cell*, 160(1-2), 191–203. 10.1016/j.cell.2014.11.041 25557079 PMC4449256

[34] Matys V., Kel-Margoulis O. V., Fricke E., Liebich I., Land S., Barre-Dirrie A., Reuter I., Chekmenev D., Krull M., Hornischer K., Voss N., Stegmaier P., Lewicki-Potapov B., Saxel H., Kel A. E., and Wingender E. (2006). TRANSFAC and its module TRANSCompel: transcriptional gene regulation in eukaryotes. *Nucleic Acids Res*., 34, D108–D110. 10.1093/nar/gkj143 16381825 PMC1347505

[35] Badis G., Berger M. F., Philippakis A. A., Talukder S., Gehrke A. R., Jaeger S. A., Chan E. T., Metzler G., Vedenko A., Chen X., Kuznetsov H., Wang C., Coburn D., Newburger D. E., Morris Q., Hughes T. R., and Bulyk M. L. (2009). Diversity and complexity in DNA recognition by transcription factors. *Science*, 324, 1720–1723. 10.1126/science.1162327 19443739 PMC2905877

[36] Ehret G. B., Reichenbach P., Schindler U., Horvath C. M., Fritz S., Nabholz M., and Bucher P. (2001). DNA binding specificity of different STAT proteins. *J. Biol. Chem*., 276, 6675–6688. 10.1074/jbc.M001748200 11053426

[37] Benjamini Y. and Hochberg Y. (1995). Controlling the False Discovery Rate: A Practical and Power Approach to Multiple Testing. *J. Roy. Stat. Soc. B*, 57(1), 289–300.

[38] Venables W. N. and Ripley B. D. (2002). Modern Applied Statistics with S. Springer, New York,Fourth Edition.

[39] Wickham H.. (2016) ggplot2: Elegant Graphics for Data Analysis. Springer-Verlag New York.

[40] Zuguang Gu Z., Gu L., Eils R., Schlesner M., Brors B. (2014) circlize implements and enhances circular visualization in R. *Bioinformatics*, 30, 2811–2812. 10.1093/bioinformatics/btu393 24930139

[41] Shannon P. and Markiel A. and Ozier O. and Baliga N.S. and Wang J.T. and Ramage D. and Amin N. and Schwikowski B. and Ideker T. (2003). Cytoscape: a software environment for integrated models of biomolecular interaction networks. *Genome Research*, 13:11, 2498–2504. 10.1101/gr.1239303 14597658 PMC403769

[42] Maglott D., Ostell J., Pruitt K. D., and Tatusova T. (2011). Entrez Gene: gene-centered information at NCBI. *Nucleic Acids Res*., 39 (suppl 1), D52–D57. 10.1093/nar/gkq1237 21115458 PMC3013746

[43] Brown G. R., Hem V., Katz K. S., Ovetsky M., Wallin C., Ermolaeva O., Tolstoy I., Tatusova T., Pruitt K. D., Maglott D. R., Murphy T. D. (2015). Gene: a gene-centered information resource at NCBI *Nucleic Acids Res*,43(Database issue), D36–42. 10.1093/nar/gku1055 25355515 PMC4383897

[44] UniProt Consortium (2017). UniProt: the universal protein knowledgebase. *Nucleic Acids Res*., 45, D158–D169. 10.1093/nar/gkw1099 27899622 PMC5210571

[45] Warde-Farley D., Donaldson S. L., Comes O., Zuberi K., Badrawi R., Chao P., Franz M., Grouios C., Kazi F., Lopes C. T., Maitland A., Mostafavi S., Montojo J., Shao Q., Wright G., Bader G. D., and Morris Q. (2010). The GeneMANIA prediction server: biological network integration for gene prioritization and predicting gene function. *Nucleic Acids Res*., 38, W214–W220. 10.1093/nar/gkq537 20576703 PMC2896186

[46] Flicek P., Amode M. R., Barrell D., Beal K., Billis K., Brent S., Carvalho-Silva D., Clapham P., Coates G., Fitzgerald S., Gil L., García Girón C., Gordon L., Hourlier T., Hunt S., Johnson N., Juettemann T., Kähäri A. K., Keenan S., Kulesha E., Martin F. J., Maurel T., McLaren W. M., Murphy D. N., Nag R., Overduin B., Pignatelli M., Pritchard B., Pritchard E., Riat H. S., Ruffier M., Sheppard D., Taylor K., Thormann A., Trevanion S. J., Vullo A., Wilder S. P., Wilson M., Zadissa A., Aken B. L., Birney E., Cunningham F., Harrow J., Herrero J., Hubbard T. J. P., Kinsella R., Muffato M., Parker A., Spudich G., Yates A., Zerbino D. R., and Searle S. M. J. (2014). Ensembl 2014. *Nucleic Acids Res*., 42, D749–D755. 10.1093/nar/gkt1196 24316576 PMC3964975

[47] Carithers Latarsha J. and Ardlie Kristin and Barcus Mary and Branton Philip A. and Britton Angela and Buia Stephen A. and Compton Carolyn C. and DeLuca David S. and Peter-Demchok Joanne and Gelfand Ellen T. and Guan Ping and Korzeniewski Greg E. and Lockhart Nicole C. and Rabiner Chana A. and Rao Abhi K. and Robinson Karna L. and Roche Nancy V. and Sawyer Sherilyn J. and Segrè Ayellet V. and Shive Charles E. and Smith Anna M. and Sobin Leslie H. and Undale Anita H. and Valentino Kimberly M. and Vaught Jim and Young Taylor R. and Moore Helen M. (2015). A Novel Approach to High-Quality Postmortem Tissue Procurement: The GTEx Project. *Biopreservation and Biobanking*, 13, 311–319. 10.1089/bio.2015.0032 26484571 PMC4675181

[48] Ernst J., Kheradpour P., Mikkelsen T. S., Shoresh N., Ward L. D., Epstein C. B., Zhang X., Wang L., Issner R., Coyne M., Ku M., Durham T., Kellis M., and Bernstein B. E. (2011). Mapping and analysis of chromatin state dynamics in nine human cell types. *Nature*, 473, 43–49. 10.1038/nature09906 21441907 PMC3088773

[49] Song L., Zhang Z., Grasfeder L. L., Boyle A. P., Giresi P. G., Lee B.-K., Sheffield N. C., Gräf S., Huss M., Keefe D., Liu Z., London D., McDaniell R. M., Shibata Y., Showers K. A., Simon J. M., Vales T., Wang T., Winter D., Zhang Z., Clarke N. D., Birney E., Iyer V. R., Crawford G. E., Lieb J. D., and Furey T. S. (2011). Open chromatin defined by DNaseI and FAIRE identifies regulatory elements that shape cell-type identity. *Genome Res*., 21, 1767 gr.121541.111. 10.1101/gr.121541.111 PMC3202292 21750106

[50] Hashimoto Tatsunori and Sherwood Richard I. and Kang Daniel D. and Rajagopal Nisha and Barkal Amira A. and Zeng Haoyang and Emons Bart J. and Srinivasan Sharanya and Jaakkola Tommi and Gifford David K. (2016). A synergistic DNA logic predicts genome-wide chromatin accessibility. *Genome Research*, 26, 1430–1440. 10.1101/gr.199778.115 27456004 PMC5052050

[51] Bieller A., Pasche B., Frank S., Gläser B., Kunz J., Witt K., and Zoll B. (2001). Isolation and Characterization of the Human Forkhead Gene FOXQ1. *Cell Biol*., 20, 555–561. 10.1089/104454901317094963 11747606

[52] Ingenuity ^®^ Systems (IPA).

[53] Whitfield T. and Wang J. and Collins P. and Partridge E. C. and Aldred S. and Trinklein N. and Myers R. and Weng Z. (2012) Functional analysis of transcription factor binding sites in human promoters. *Genome Biology*, 13, R50+ 10.1186/gb-2012-13-9-r50 22951020 PMC3491394

[54] Ravasi T., Suzuki H., Cannistraci C. V. V., Katayama S., Bajic V. B., Tan K., Akalin A., Schmeier S., Kanamori-Katayama M., Bertin N., Carninci P., Daub C. O., Forrest A. R., Gough J., Grimmond S., Han J.-H. H., Hashimoto T., Hide W., Hofmann O., Kamburov A., Kaur M., Kawaji H., Kubosaki A., Lassmann T., van Nimwegen E., MacPherson C. R. R., Ogawa C., Radovanovic A., Schwartz A., Teasdale R. D., Tegnér J., Lenhard B., Teichmann S. A., Arakawa T., Ninomiya N., Murakami K., Tagami M., Fukuda S., Imamura K., Kai C., Ishihara R., Kitazume Y., Kawai J., Hume D. A., Ideker T., and Hayashizaki Y. (2010). An atlas of combinatorial transcriptional regulation in mouse and man. *Cell*, 140, 744–752. 10.1016/j.cell.2010.01.044 20211142 PMC2836267

[55] Chatraryamontri A., Breitkreutz B.-J., Heinicke S., Boucher L., Winter A., Stark C., Nixon J., Ramage L., Kolas N., O’Donnell L., Reguly T., Breitkreutz A., Sellam A., Chen D., Chang C., Rust J., Livstone M., Oughtred R., Dolinski K., and Tyers M. (2013). The biogrid interaction database: 2013 update. *Nucleic Acids Res*., 41, D816–D823. 10.1093/nar/gks1158 23203989 PMC3531226

[56] Lynn D. J., Winsor G. L., Chan C., Richard N., Laird M. R., Barsky A., Gardy J. L., Roche F. M., Chan T. H., Shah N., Lo R., Naseer M., Que J., Yau M., Acab M., Tulpan D., Whiteside M. D., Chikatamarla A., Mah B., Munzner T., Hokamp K., Hancock R. E., and Brinkman F. S. (2008). InnateDB: facilitating systems-level analyses of the mammalian innate immune response. *Mol. Syst. Biol*., 4 10.1038/msb.2008.55 18766178 PMC2564732

[57] Chen X., Xu H., Yuan P., Fang F., Huss M., Vega V. B., Wong E., Orlov Y. L., Zhang W., Jiang J., Loh Y.-H. H., Yeo H. C. C., Yeo Z. X. X., Narang V., Govindarajan K. R. R., Leong B., Shahab A., Ruan Y., Bourque G., Sung W.-K. K., Clarke N. D., Wei C.-L. L., and Ng H.-H. H. (2008a). Integration of external signaling pathways with the core transcriptional network in embryonic stem cells. *Cell*, 133, 1106–1117. 10.1016/j.cell.2008.04.043 18555785

[58] Pardo M., Lang B., Yu L., Prosser H., Bradley A., Babu M. M., and Choudhary J. (2015). An Expanded Oct4 Interaction Network: Implications for Stem Cell Biology, Development, and Disease. *Cell Stem Cell*, 6, 382–395. 10.1016/j.stem.2010.03.004 PMC2860244 20362542

[59] Vella P., Barozzi I., Cuomo A., Bonaldi T., and Pasini D. (2012). Yin yang 1 extends the myc-related transcription factors network in embryonic stem cells. *Nucleic Acids Res*., 40, 3403–3418. 10.1093/nar/gkr1290 22210892 PMC3333890

[60] Weiss M. J. and Orkin S. H. (1995). GATA transcription factors: key regulators of hematopoiesis. *Exp. hematol*., 23, 99–107. 7828675

[61] Festuccia N., Osorno R., Halbritter F., Karwacki-Neisius V., Navarro P., Colby D., Wong F., Yates A., Tomlinson S. R., and Chambers I. (2012). Esrrb Is a Direct Nanog Target Gene that Can Substitute for Nanog Function in Pluripotent Cells. *Cell Stem Cell*, 11, 477–490. 10.1016/j.stem.2012.08.002 23040477 PMC3473361

[62] Martello G., Sugimoto T., Diamanti E., Joshi A., Hannah R., Ohtsuka S., Göttgens B., Niwa H., and Smith A. (2012). Esrrb Is a Pivotal Target of the Gsk3/Tcf3 Axis Regulating Embryonic Stem Cell Self-Renewal. *Cell Stem Cell*, 11, 491–504. 10.1016/j.stem.2012.06.008 23040478 PMC3465555

[63] Heinz S., Benner C., Spann N., Bertolino E., Lin Y.C., Laslo P., Cheng J.X., Murre C., Singh H., and Glass C.K. (2010). Simple combinations of lineage determining transcription factors prime cis-regulatory elements required for macrophage and B cell identities. *Mol. Cell*, 38, 576–589. 10.1016/j.molcel.2010.05.004 20513432 PMC2898526

