# Supplementary material for: coTRaCTE predicts co-occurring transcription factors within cell-type specific enhancers
Source: PLoS Comput Biol. 2018 Aug 24;14(8):e1006372. doi: 10.1371/journal.pcbi.1006372 (PMC6126874; doi:10.1371/journal.pcbi.1006372)
Supplement: S1 Appendix — (PDF) [file pcbi.1006372.s001.pdf]

coTRaCTE predicts co-occurring transcription factors within cell-type specific enhancers

Alena van Bömmel[1], Michael I. Love[2], Ho-Ryun Chung[3], and Martin Vingron[1]

[1]Department of Computational Molecular Biology, Max Planck Institute for Molecular Genetics, Ihnestr. 63-73, D-14195, Berlin, Germany
[2]Department of Biostatistics, Department of Genetics, University of North Carolina at Chapel Hill, 135 Dauer Drive, Chapel Hill, NC
[3]Otto Warburg Laboratory, Max Planck Institute for Molecular Genetics, Ihnestr. 63-73, D-14195, Berlin, Germany

Supporting information

Selection of parameters for TF motif overrepresentation in CTS-DHSs

To find overrepresented TF motifs in CTS-DHSs, the following two parameters l defining the most cell-type specific and most ubiquitous DHSs which are ranked in the ranked list representation of each TF, and parameter k selecting the top-ranked DHSs must be specified.
To test the consistency of the enriched transcription factors in the CTS-DHSs we calculated Fisher's exact test for all possible combinations of thresholds $k \in \{500, 1000, 2000\}$ and $l \in \{1000, 2000, 5000, 7000\}$. The dependency of the log_{10} p-value on the choice of different thresholds for all 554 TF motifs in 3 different cell types is shown in S1 Fig. In general, we can observe a decline of the significance with decreasing number of selected CTS-DHSs (l) and

with decreasing number of selected top-scored DHSs (k). However, this trend is relatively weak in the majority of studied cell types as shown for leukemia and B-lymphocyte in S1 Fig.

In addition, the top-20 enriched motifs selected with $k = 500$ and $l = 5000$ were highlighted in red and the selection of the top-20 enriched motifs with $k = 1000$ and $l = 1000$ was highlighted in blue. The first combination of thresholds $k = 500$; $l = 5000$ represents one extreme case when k is relatively small and l large. The second combination of the parameters with $k = 1000$ and $l = 1000$ corresponds to the case when only the most cell-type specific DHSs are selected and the top-ranked DHSs account for half of the list (since the length of the list is $2l$).

For many cell types, the agreement of the top-20 enriched motifs between these two extremes is very large and is consistent over the majority of the threshold combinations, as depicted in the case of leukemia (see S1 Fig(a)). However, there are several cell types (mainly the embryonic stem cells, see S1 Fig(b)) where the agreement between the two selections of thresholds is very small. The top-ranked motifs based on $k = 1000$ and $l = 1000$ have lower significance for other combinations of thresholds with $l > 1000$ and the top-ranked motifs based on $k = 500$ and $l = 5000$ have smaller significance for $l \leq 2000$. For some of the cell types one can observe a sort of mixture of the two above cases; relative consistency over all threshold combinations with few outliers which show low significance in one of the extreme combination of thresholds, as shown in the case of B-lymphocyte in S1 Fig(c).

After evaluation of all cell types, the combination $k = 500$ and $l = 5000$ was selected for the further analysis of the overrepresented TF motifs. First, the biological interpretation of this choice is reasonable, 5000 of the most cell-type specific accessible regions in the genome are analyzed and our focus is on the very top of the ranked lists. Second, this combination of thresholds does not overestimate the significance as for other combinations (e.g. $k \in \{1000, 2000\}$ and $l \in \{5000, 7000\}$, respectively) where many of the TF motifs reach the minimal possible p-value of 10^{-150}. With these selected cutoffs of $k = 500$ and $l = 5000$ the significance for all contingency tables corresponding to all possible combinations of TFs and cell types was calculated.

Parameter choice for predicting co-occurring TFs on CTS-DHSs

The L_{ct} score depends on two threshold parameters k_1 and k_2 defining the top-ranked sites for the first and second TF in the pair, respectively. In addition, the construction of the partial contingency tables depends on the choice of parameter l which selects the most cell-type specific sites and most ubiquitous sites.

To investigate the stability of the obtained results, we calculated the L_{ct} scores for all TF pairs for different combinations of parameters. Then TF pairs with the largest L_{ct} scores ($L > 99.5\%$-quantile) were selected and studied for the consistency of obtained results. For easier comparison, thresholds k_1 and k_2 were chosen to be equal in all combinations; all pairs of TF motifs were mapped to the corresponding TF names or groups.

Two matrices with numbers of identical TF pairs predicted with various combinations of parameters $k_1 = k_2$ and l in embryonic stem cells (ESCs) and in B-lymphocytes are shown in S2 Fig. Both matrices show very similar trend: with the choice of $l \in \{1000, 2000, 5000\}$ the choice of k_1 and k_2 is not important for the group of highly significant TF pairs; the predicted TF pairs remain exactly the same for different values of $k_1 = k_2$ (see dark blue boxes along the diagonal in S2 Fig). Setting the threshold $l = 7000$ causes a high variability between the significant TF pairs defined with different thresholds $k_1 = k_2 \in \{500, 1000, 2000\}$. Further, there is a high similarity of the predictions derived with $l = 2000$ and $t = 1000$ with different values of $k_1 = k_2$. The agreement between the two sets of significant TF pairs is 102 out of 232 and 269 TF pairs (in B-lymphocytes, S2 Fig(b)) and 70 out of 215 and 233 TF pairs (in ESCs, S2 Fig(a)). The combination of thresholds $l = 7000$ and $k_1 = k_2 = 2000$ gives very similar results to threshold $l = 5000$ with various values for $k_1 = k_2$. This behavior can be observed for the majority of studied cell types.

These results suggest that the strongest cell-type specific signal is among the top 5000 CTS-DHSs, since for this threshold $l \leq 5000$ highly consistent results were obtained. For further analysis, we use the longest possible list of

CTS-DHSs and ubiquitous DHSs with $l = 5000$ and choose as appropriate threshold $k_1 = k_2 = 1000$.

Overrepresented TFs in CTS-DHSs have cell-type specific functions

The overrepresented TF motifs on CTS-DHSs were investigated for their known functions in the corresponding cell types. The results for all TFs are visualized in a color heatmap in S4 Fig where each cell indicates the significance of the Fisher's exact test fof each TF in the corresponding cell type. For simplification, the general TFs and TFs which did not show high significance ($-\log_{10}(p\text{-value}) < 20$) in any of the cell types were removed, resulting in 192 TFs showed in all 64 cell types. First, a group of TFs such as CREB1, ATF, EVI1, EGR and LHX3 factors show significant enrichment in many cell types (mainly in various fibroblasts, endothelial and epithelial cell lines). A large group of TFs including FOX proteins, POU proteins, HNF proteins and CEBP proteins is enriched in fibroblasts.

Further, we can identify several groups of factors overrepresented in specific cell types. For example, TFs overrepresented in cell types of the immune system (B-lymphocyte, T-cell, primary T-cell, regulatory T-cell, monocyte, hematopoietic progenitor cell, marrow stromal cells and leukemia) are known regulators of immunological functions such as ATF family members (with ATF, JUN, FOS), ETS family members (with ELF, ELK, ETS, FLI1, GABPA, SPI1), EGR proteins, IRF proteins, GATA proteins, and BACH proteins [1,2]. Three of the top-100 factors in B-lymphocyte (E2F, PRDM1, ZNF384) were found as associated TFs with so-called high plasticity regions in B-lymphocytes by [3]. Our findings of enriched TFs in monocytes such as: ETS, ATF, IRF, SPI/KLF and CEBP are in agreement with previous studies in monocytes [4,5]. Concerning the enriched factors in leukemia, many of them such as LMO2, GATA factors and EVI1 were identified in previous studies [6,7] to have an important function in myeloid leukemia. Further, ETS1, HIF1A and GATA1 are regulators of myeloid cell differentiation [8,9].

Looking at the muscle-related cell types (skeletal muscle myoblasts, skeletal

striated muscle cells, cardiac myocytes and brain vascular smooth muscle cells) we find the overrepresented TFs MYF, MYOD, MYOG, NFYA and TCF3 which are known regulators in muscle cells.

Overrepresented TFs in cell types originating from lung tissue (fetal lung fibroblast, embryonic lung fibroblast, lung fibroblast and pulmonary fibroblast) are members of the FOX family and of the ETS family. Several of these factors are known regulators in lung development and lung morphogenesis, (ETS1, FOXA, FOXF1 and FOXL1) [1, 10]. Many FOX family members are enriched in the embryonic lung fibroblast and the fetal lung fibroblast which is in agreement with previous studies which found FOX genes involved in embryonic development [8, 9]. The most overrepresented factors in the above mentioned cell types are summarized in S1 Table.

Co-occurring TFs in ubiquitous DHSs include several promoter-specific TFs

As a contrast to cell-type specific co-occurring TFs, we predicted TF pairs that co-occur on the ubiquitous DHSs more likely than on the cell-type specific DHSs, e.g. TF pairs with largest negative L_l scores over majority of cell types. The main regulators with most TF partners in the network are factors SP1, E2F and PAX factors. Furthermore, three small subnetworks with STAT factors, GABPA and NKX2-5 factor, a subnetwork with JUN:FOS central hub, and a small subnetwork with NFY central hub are presented in the network (see S6 Fig). Several hubs, such as E2F1, NFY, SP1, STAT factors together with other presented factors in the network (ATF, CREB, NRF1, TBP) were described in previous studies [11, 12] as promoter-specific or promoter-centric TFs. This is in agreement with our predictions since the ubiquitous DHSs overlay in large part promoter regions.

Co-occurring TF pairs in immune-specific DHSs

We investigated the predicted co-occurring TF pairs in immune cells, such as hematopoietic progenitor cells (HPCs), B-lymphocyte, T-cell (primary and regulatory), monocyte and leukemia. First, we analyzed the expression

of the TFs among the co-occurring TF pairs in the corresponding cell type and whether these TFs have a known regulatory function in hematopoiesis. Approximately one quarter of the factors (from 21% in B-lymphocytes to 38% in monocytes) are known regulators in hematopoietic differentiation or other immunological function [1, 2, 13, 14]. Notably, more than three quarters of all predicted TFs (from 75% in T-cells to 80% in HPCs) are expressed in immune cells, when comparing with the RNA-seq data from [15]. One has to point out that all these factors were selected *only* by the high L_{ct} score without any knowledge of their possible function or expression in the corresponding cell type. Further, we provide a detailed analysis of the predicted co-occurring TF pairs in two cell types: hematopoietic progenitor cells and in K562 cells (leukemia). To focus on the cell-type-specific TF co-occurrence, we removed all general significant TF pairs which appear in 30 or more cell lines and construct regulatory networks from all significant TF pairs in the particular cell type.

The regulatory network in the HPCs consists then of 178 edges (TF pairs) among 120 nodes (TFs) and is dominated by a highly connected large subnetwork followed by several triplets and pairs of co-occurring TFs. TFs with most co-occurring partners in the network are EVI1, GATA, POU6F1, ONECUT and TEF. Among them, EVI1 and GATA are known important regulators of white blood progenitor cells and the homeodomain proteins POU6F1 and ONECUT are known regulators of pluripotency and differentiation [8, 9, 16] which are important in the hematopoiesis, too. Several key hematopoietic regulators such as: TCF3, LEF1, IKZF1, IRF1 and bHLH-binding proteins SREBF and USF [1, 2, 13, 14] are present also in the network (see S10 FigA, known regulators of hematopoiesis are highlighted as rectangles with yellow borders). Further, the predicted network in hematopoietic progenitor cells includes several experimental validated protein-protein interactions (PPIs) [17, 18] such as the known complex of NFKB/RELA and STAT3, GATA:MEF2A, GATA:POU1F1, CEBP:ATF/CREB and CEBP:STAT5A (red edges in S10 Fig).

For comparison, let us investigate the transcriptional network derived for cancer cell line K562, see S10 FigB. The network consists of 184 edges (TF

pairs) among 121 nodes (TFs) and is dominated by a large subnetwork of highly connected TFs. The most connected TFs are distinct from those in hematopoietic progenitor cells, namely: CDX, HNF4A, PBX1, LHX3 and FOXF1:FOXJ1. Among these, PBX1 (Pre-B-Cell Leukemia Homeobox 1) is associated with pre-B-cell acute lymphoblastic leukemia [19], CDX1 inhibits T-cell factor transcriptional activity and CDX4 is involved in hematopoiesis. Functions of FOXF1 and FOXJ1 is not well studied yet, however they may play a role in the embryonic development [8, 9]. Interestingly, the network in leukemia includes several tumor-related TFs such as TAL1, LMO2 and STAT5 that are known transcriptional regulators in leukemia [9,20,21]. These factors are not predicted among the co-occurring TFs in the hematopoietic progenitor cells. Further, we predicted several co-occurring TF pairs involving GATA proteins, such as: GATA:LMO2; and GATA:EVI1. This result is in agreement with previous studies which shown that GATA proteins play a very important role in the transcriptional regulation of leukemia [22]. Further, several co-occurring TF pairs predicted with our method are known experimental PPIs: FOX:TBP, ATF/CREB: STAT5A, MAF and ETS-binding proteins, ESR:NR2F1 and the complex STAT3:NFKB1:RELA.

S1 Fig. Dependency of the significance in Fisher's exact test on threshold selection for 477 TF motifs in three different cell lines. Dependency of the significance in Fisher's exact test on threshold selection for 477 TF motifs in (a) leukemia, (b) embryonic stem cells (ESCs) and (c) B-lymphocytes. The significance is represented as $-\log_{10}$ p-value on the vertical axis. 11 combinations of thresholds k (defining the top-ranked DHSs) and t (defining the number of cell-type specific and ubiquitous DHSs) is depicted on the horizontal axis. Top-20 enriched TF motifs selected with two extreme values of k and t are highlighted in red and blue, respectively.

S2 Fig. Consistency of the most significant TF pairs on CTS-DHSs for different combinations of parameters. Consistency of the most significant TF pairs on CTS-DHSs for different combinations of parameters in a) embryonic stem cell (ESC) and b) B-lymphocyte. The matrix entries denote the number of identical TF pairs with the highest L_l score for 11 combinations of thresholds $k_1 = k_2$ (the first number) and of threshold t (second number).

S3 Fig. Genomic distribution of the top 5000 CTS-DHSs and of the top 5000 ubiquitous DHSs Genomic distribution of the 5000 most cell-type specific DNase hypersensitive sites in 64 cell types and of the top 5000 ubiquitous DNase hypersensitive sites sorted by the overlap with promoter regions.

S4 Fig. Overrepresented transcription factors over 64 cell types. Each cell in the matrix indicates the significance of the association between the cell type and the corresponding TF. TFs overrepresented in the majority of cell types are highlighted in red. Cell type-specific TFs are marked with boxes of color corresponding to the tissue.

S5 Fig. Network of highly frequent TF pairs predicted in at least 30 out of 64 cell types. Nodes in the network represent transcription

factors, edges are drawn between the co-occurring TF pairs predicted by CoTRaCTE. Red edges are known protein-protein interactions.

S6 Fig. Network of co-occurring TF pairs in ubiquitous DHSs. Nodes in the network represent transcription factors, edges are drawn between the co-occurring TF pairs predicted by CoTRaCTE. Red edges are known protein-protein interactions. Known promoter-specific regulators are highlighted as rectangles with red border; green nodes are TFs indicated as overrepresented on promoter sequences in [23].

S7 Fig. Comparison of co-occurring TF pairs in undifferentiated and differentiated embryonic stem cells. Comparison of predicted regulators in undifferentiated and differentiated embryonic stem cells. For each TF, the barplot shows the number of distinct co-occurring partners in undifferentiated ESCs (red) and in differentiated ESCs (blue) and the number of shared co-occurring partners on both cell lines (black). The left column shows the absolute numbers, the right column shows the proportions.

S8 Fig. Heatmap of overlapping predicted co-occurring TF pairs on ubiq-DHSs over 64 cell types. Each cell depicts the number of TF pairs shared between the corresponding pair of cell types.

S9 Fig. Boxplots showing the distributions of GC-content in the ubiq-DHSs and CTS-DHSs by cell type. Each boxplot shows the GC-content distribution of the 5000 most cell-type specific and most ubiquitous DHSs, respectively. The boxes of each cell type are coloured by the corresponding tissue. Blue line depicts the average GC content of the human genome (hg19) which is 40.9%.

S10 Fig. Network of predicted co-occurring TF pairs in hematopoietic progenitor cells and leukemia. A) Network of predicted co-occurring TFs in hematopoietic progenitor cells. Nodes in the network represent transcription factors, edges are drawn between co-occurring TF pairs predicted

by coTRaCTE. Red edges are known protein-protein interactions which are also predicted by coTRaCTE. TFs expressed in the cell line are highlighted in green; darker tone indicates stronger evidence of expression in related cell types. Known regulators in hematopoiesis are highlighted as rectangles with yellow border. Node size reflects the number of predicted co-occurring TF partners.

B) Network of predicted co-occurring TFs in leukemia.

S1 Table. Most significant cell-type specific TFs in various cell types. TFs in bold are known transcription regulators in the corresponding cell type.

S2 Table. Top-10 predicted TF-TF dimers by Jankowski *et al.* [24] compared to predictions of coTRaCTE. Predicted TF-TF dimers by [24] with the predicted cell type (first two columns); predicted co-occurring TF pairs by coTRaCTE including the predicted cell type (third and fourth column) and literature evidence (fifth column).

S1 File. DNase-seq data from ENCODE project used in the analysis.

S2 File. List of 554 TF motifs and corresponding TF groups. List of all 554 TF motifs from TRANSFAC database used for the analysis and their corresponding TF names(groups). Table includes an alternative factor name and further information from TRANSFAC about the availability and quality of the position weight matrix (PWM).

S3 File. Predicted co-occurring TF pairs in 64 cell types Predicted co-occurring TF pairs in separate files for each cell type. The columns show the following: TF 1 name, TF 2 name, $-\log_{10}(p-\text{value}_{\text{CTS}})$, $-\log_{10}(p-\text{value}_{\text{ubiq}})$, L_l score, frequency of the TF pair in other cell types, known PPI (1=yes, 0=no) , motif similarity defined in [37].

S4 File. Predicted co-occurring TF pairs by CoTRaCTE and by ENCODE. Predicted co-occurring TF pairs that are comparable with the ENCODE predictions. The columns show the following: TF 1 name, TF 2 name, prediction by ENCODE, known PPI (1=yes, 0=no), Ensembl ID1, Ensembl ID2 and other experimental evidence.

Supplementary References

1. Matys V, Kel-Margoulis OV, Fricke E, Liebich I, Land S, Barre-Dirrie A, Reuter I, Chekmenev D, Krull M, Hornischer K, Voss N, Stegmaier P, Lewicki-Potapov B, Saxel H, Kel AE, Wingender E. TRANSFAC and its module TRANSCompel: transcriptional gene regulation in eukaryotes. Nucleic Acids Res. 2006;34:D108–D110.

2. Nutt SL, Kee BL. The Transcriptional Regulation of B Cell Lineage Commitment. Immunity. 2007;26:715–725.

3. Pinello L, Xub J, Orkin SH, Yuan GC. Analysis of chromatin-state plasticity identifies cell-type-specific regulators of H3K27me3 patterns. Proc Natl Acad Sci U S A. 2014;111(3):E344–E353.

4. Martens JHA, Rico D, Downes K, Richardson D, Breschi A, Heath S, et al. Epigenomic plasticity of human neutrophils and monocytes in cord and peripheral blood. submitted. 2014;.

5. Huber R, Pietsch D, Günther J, Welz B, Vogt N, Brand K. Regulation of monocyte differentiation by specific signaling modules and associated transcription factor networks. Cell Mol Life Sci. 2014;71:63–92.

6. Tenen DG, Hromas R, Licht JD, Zhang DE. Transcription Factors, Normal Myeloid Development, and Leukemia. Blood. 1997;90:489–519.

7. Nucifora G, Laricchia-Robbio L, Senyuk V. EVI1 and hematopoietic disorders: history and perspectives. Gene. 2006;368:1–11.

8. Maglott D, Ostell J, Pruitt KD, Tatusova T. Entrez Gene: gene-centered information at NCBI. Nucleic Acids Res. 2011;39 (suppl 1):D52–D57.

9. Brown GR, Hem V, Katz KS, Ovetsky M, Wallin C, Ermolaeva O, Tolstoy I, Tatusova T, Pruitt KD, Maglott DR, Murphy TD. Gene: a gene-centered information resource at NCBI. Nucleic Acids Res. 2015;43(Database issue):D36–42.

10. Maeda Y, Dave V, Whitset JA. Transcriptional control of lung morphogenesis. Physiol Rev. 2007;87:219–244.

11. Whitfield T,Wang J, Collins P, Partridge EC, Aldred S, Trinklein N, Myers R, Weng Z. Functional analysis of transcription factor binding sites in human promoters. Genome Biology. 2012;13:R50+.

12. Neph S, Vierstra J, Stergachis AB, Reynolds AP, Haugen E, Stamatoyannopoulos JA, et al. An expansive human regulatory lexicon encoded in transcription factor footprints. Nature. 2012;489:83–90.

13. Ramirez J, Lukin K, Hagman J. From hematopoietic progenitors to B cells: mechanisms of lineage restriction and commitment. Current Opinion in Immun. 2010;22:177–184

14. Wilson NK, Foster SD, Wang X, Knezevic K, Schütte J, Kaimakis P, Chilarska PM, Kinston S, Ouwehand WH, Dzierzak E, Pimanda JE, de Bruijn MF, Göttgens B. Combinatorial transcriptional control in blood stem/progenitor cells: genome-wide analysis of ten major transcriptional regulators. Cell Stem Cell. 2010;7(4):532–44.

15. Flicek P, Amode MR, Barrell D, Beal K, Billis K, Searle SMJ, et al. Ensembl 2014. Nucleic Acids Res. 2014;42:D749–D755.

16. UniProt Consortium. UniProt knowledgebase: a hub of integrated protein data. Database. 2011.

17. Chatraryamontri A, Breitkreutz BJ, Heinicke S, Boucher L, Winter A, Stark C, Nixon J, Ramage L, Kolas N, O'Donnell L, Reguly T, Breitkreutz A, Sellam A, Chen D, Chang C, Rust J, Livstone M, Oughtred R, Dolinski K, Tyers M. The biogrid interaction database: 2013 update. Nucleic Acids Res. 2013;41:D816–D823.

18. Ravasi T, Suzuki H, Cannistraci CVV, Katayama S, Bajic VB, Hayashizaki Y. An atlas of combinatorial transcriptional regulation in mouse and man. Cell. 2010; 140:744–752.

19. Van Dijk MA, Voorhoeve PM, Murre C. Pbx1 is converted into a transcriptional activator upon acquiring the N-terminal region of E2A in pre-B-cell acute lymphoblastoid leukemia. Proc Natl Acad Sci U S A. 1993; 90(13):6061–5

20. Palomero T, Odom DT, O'Neil J, Ferrando AA, Margolin A, Neuberg DS, Winter SS, Larson RS, Li W, Liu XS, Young RA, and Look AT. Transcriptional regulatory networks downstream of TAL1/SCL in T-cell acute lymphoblastic leukemia. Blood. 2006; 108:986–992

21. Cleveland SM, Goodings C,Tripathi RM, Elliott N, Thompson MA, Guo Y, Shyr Y, Davé UP. LMO2 induces T-cell leukemia with epigenetic deregulation of CD4. Experimental Hematology. 2014; 42(7):581–593.e5

22. Hou Q,Liao F, Zhang S, Zhang D, Zhang Y, Zhou X, Xia X, Ye Y, Yang H, Li Z, Wang L, Wang X, Ma Z, Zhu Y, Ouyang L, Wang Y, Zhang H, Yang L, Xu H, Shu Y. Regulatory network of GATA3 in pediatric acute lymphoblastic leukemia. Oncotarget. 2017; 8(22):36040–36053

23. Myšičková A, Vingron M. Detection of interacting transcription factors in human tissues using predicted DNA binding affinity. BMC Genomics. 2012;13 Suppl 1:S2+.

24. Jankowski A, Szczurek E, Jauch R, Tiuryn J, Prabhakar S. Comprehensive prediction in 78 human cell lines reveals rigidity and compactness of transcription factor dimers. Genome Res. 2013;23:1307–1318.

25. De Masi F, Grove CA, Vedenko A, Alibés A, Gisselbrecht SS, Serrano L, et al. Using a structural and logics systems approach to infer bHLH-DNA binding specificity determinants. Nucleic Acids Res. 2011;39:4553–4563.

26. Tanaka N, Kawakami T, Taniguchi T. Recognition DNA sequences of interferon regulatory factor 1 (IRF-1) and IRF-2, regulators of cell growth and the interferon system. Mol Cell Biol. 1993;13:4531–4538.

27. Treiber N, Treiber T, Zocher G, Grosschedl R. Structure of an Ebf1:DNA complex reveals unusual DNA recognition and structural homology with Rel proteins. Genes & Development. 2010;24:2270–2275.

28. Chen X, Xu H, Yuan P, Fang F, Huss M, Vega VB, Wong E, Orlov YL, Zhang W, Jiang J, Loh YH, Yeo HC, Yeo ZX, Narang V, Govindarajan KR, Leong B, Shahab A, Ruan Y, Bourque G, Sung WK, Clarke ND, Wei CL, Ng HH. Integration of external signaling pathways with the core transcriptional network in embryonic stem cells. Cell. 2008; 133:1106–1117.

29. Wang D, Garcia-Bassets I, Benner C, Li W, Su X, Zhou Y, et al. Reprogramming transcription by distinct classes of enhancers functionally defined by eRNA. Nature. 2011;474:390–394.

30. Courtois G, Baumhueter S, Crabtree GR. Purified hepatocyte nuclear factor 1 interacts with a family of hepatocyte-specific promoters. Proc Natl Acad Sci U S A. 1988;85:7937–7941.

31. Cheret C, Doyen A, Yaniv M, Pontoglio M. Hepatocyte nuclear factor 1 alpha controls renal expression of the Npt1-Npt4 anionic transporter locus. J Mol Biol. 2002;322:929–941.

32. Friedman PN, Chen X, Bargonetti J, Prives C. The p53 protein is an unusually shaped tetramer that binds directly to DNA. Proceedings of the National Academy of Sciences. 1993;90:3319–3323.

33. McLure KG, Lee PWK. How p53 binds DNA as a tetramer. The EMBO Journal. 1998;17:3342–3350.

34. Kahler RA, Westendorf JJ. Lymphoid Enhancer Factor-1 and β-Catenin Inhibit Runx2-dependent Transcriptional Activation of the Osteocalcin Promoter. J Biol Chem. 2003;278:11937–11944.

35. Reinhold MI, Naski MC. Direct Interactions of Runx2 and Canonical Wnt Signaling Induce FGF18. J Biol Chem. 2007;282:3653–3663.

36. Hollenhorst PC, Chandler KJ, Poulsen RL, Johnson WE, Speck NA, Graves BJ. DNA Specificity Determinants Associate with Distinct Transcription Factor Functions. PLoS Genet. 2009;5:e1000778+.

37. Pape UJ, Rahmann S, Vingron M. Natural similarity measures between position frequency matrices with an application to clustering. Bioinformatics. 2008;24:350–357.
