# Supplementary material for: coTRaCTE predicts co-occurring transcription factors within cell-type specific enhancers
Source: PLoS Comput Biol. 2018 Aug 24;14(8):e1006372. doi: 10.1371/journal.pcbi.1006372 (PMC6126874; doi:10.1371/journal.pcbi.1006372)
Supplement: S1 Table — TFs in bold are known transcription regulators in the corresponding cell type. (PDF) [file pcbi.1006372.s012.pdf]

Cell type	cell group	Associated TFs
B-lymphocyte	immune	**FOS:JUN, IRF, OCT:POU2F, TCF3, ZEB1**
T-cell	immune	BACH1, **BACH2, FOS:JUN, NKX3-1, SRY, STAT3**
T-cell primary	immune	BACH1, **BACH2, FOS:JUN, NFE2, NFYA**
T-cell regulatory	immune	**ATF, BACH1, BACH2, FOS:JUN, LHX3, NFE2, NFYA, ZEB1**
hematopoietic progenitor	immune	**FOS:JUN, GATA, MYOD, NFYA, TCF3, ZEB1**
marrow stromal	immune	AHR, LHX3, NKX3-1, **POU1F1, POU3F2, POU6F1**
monocyte	immune	**CEBP, ELF:ELK:ETS:FLI1:GABP, HIF1A, IRF, SPI1, STAT5A**
leukemia	immune cancer	AHR, **ATF, EVI1, ETS1:P54, GATA1, GATA3, GATA6, HIF1A, LMO2**
brain vascular smooth muscle	muscle	ETS1, FOX, **NFYA, SRY**
cardiac myocytes	muscle	AHR, CEBP, EVI1, GATA, HMGA, **NFYA, PAX4, STAT5A**
muscle myoblast	muscle	AHR, ETS1, **MEF2A, MYOD, MYOG, TCF3, TFAP4**
skeletal myoblasts	muscle	**MYOD, MYOG , TAL1:TCF, TFAP4**
skeletal striated muscle	muscle	AHR:ARNT, AR, CEBP, ETS1, HIF1A, NKX3-1, NR3C1, PATZ1, PGR, POU1F1
fetal lung fibroblast	lung	**ETS1, FOXF1, FOXI1, FOXJ2, FOXL1, FOXQ1, NKX3-1, POU1F1, TBP, TEF**
embryonic lung fibroblast	lung	**FOXA, FOXF1, FOXI1, FOXJ2, FOXL1, FOXQ1, NKX3-1, PATZ1, TBP**
lung fibroblast	lung	**ETS1, EVI1, GATA, GATA6, HIF1A, LHX3, MYCN, NKX3-1, TFAP2C, USF2**
pulmonary fibroblast	lung	CEBP, EGR4, **ETS1, FOXF1, FOXJ2, FOXL1, FOXQ1, NKX3-1, POU6F1, TBP**
ESC	stem cell	ATF, BACH1, BACH2, CREM, ETS1, FOS, JUN, MYF:MYOD:TCF, **OCT4, ZEB1**
undifferentiated ESCs	stem cell	AHR, ATF, BACH2, CREM, FOS, JUN, **NANOG, OCT4, SOX2**
differentiated ESCs	stem cell	ATF, BACH1, BACH2, CREM, ETS1, FOS, JUN, GATA1, NFE2
