# Supplementary material for: coTRaCTE predicts co-occurring transcription factors within cell-type specific enhancers
Source: PLoS Comput Biol. 2018 Aug 24;14(8):e1006372. doi: 10.1371/journal.pcbi.1006372 (PMC6126874; doi:10.1371/journal.pcbi.1006372)
Supplement: S2 Table — Predicted TF-TF dimers by [17] with the predicted cell type (first two columns); predicted co-occurring TF pairs by coTRaCTE including the predicted cell type (third and fourth column) and literature evidence (fifth column). (PDF) [file pcbi.1006372.s013.pdf]

Predicted TF-TF dimer	Predicted cell line	Predicted co-occurring TF pair	Predicted cell type	Previous studies
YY1/YY2:PUR1/GR/MEIS1	retinoblastoma	YY1:MEIS1	lung fibroblast, muscle myoblast, amniotic epithelial cells	E-box dimer (non-tissue-specific) [18]
E2F1/IRF8:IRF8/IRF1/NFAT3	B lymphocytes	E2F1/IRF8:NFAT3	fibroblasts, HPCs	IRF homotypic dimer (non-specific) [22]
STAT1/MZF1/Helios A:IKZF/Helios A	neuroblastoma	STAT1:IKZF	astrocytes, mesenchymal stem cells,ligament fibroblast	EBF1 dimer (B-lymphocytes) [23]
OCT1/OCT2/POU5F1: NANOG/SMAD1/SOX4	ESCs	OCT:SOX	ESCs	OCT-SOX heterodimer (ESCs) [37]
HNF3A:GR	prostate adenocarcinoma	FOXA1:AR	ubiquitous	FOXA1-AR heterodimer (prostate adenocarcinoma) [24]
CRX/IPF1:CRX/IPF1	kidney epithelial cells	not possible (homodimer)		HNF1 homodimer (liver and kidney cells) [26], [27]
YY1/YY2:YY1/YY2/REX1/NF1	retinoblastoma, neuroblastoma, fibroblast cells	not possible (homodimer)		E-box dimer (non-specific) [18]
p53:p53	epithelial cells	not possible (homodimer)		p53 homotetramer (non-specific) [28,29]
CBF:TCF3/TCF4/LEF1	T-lymphocytes	not predicted		RUNX-TCF heterodimer (osteoblasts) [30,32]
ETS1/ELF5:CBF	T-lymphocyte	not predicted		ETS-RUNX heterodimer (T-lymphocyte) [33]
